# Fisetin-loaded Nanoemulsion and Fecal Microbiome Extract Enhance In Vitro Inhibition of Non-Small Cell Lung Cancer Progression

**DOI:** 10.1007/s12013-025-01981-2

**Published:** 2026-01-12

**Authors:** Adam Haysom-McDowell, Keshav Raj Paudel, Samir Mehndiratta, Manisha Singh, Md. Zubbair Malik, Sofia Kokkinis, Prisca Deviani Pakan, Frederick E. Williams, Sachin Kumar Singh, Kamal Dua, Gabriele De Rubis

**Affiliations:** 1 Discipline of Pharmacy, Graduate School of Health, University of Technology Sydney, Ultimo, NSW 2007 Australia; 2 Woolcock Institute of Medical Research, Macquarie University, Sydney, NSW 2109 Australia; 3 Centre for Inflammation Centenary Institute and University of Technology Sydney, Faculty of Science, School of Life Sciences, Sydney, NSW 2007 Australia; 4 Uttaranchal Institute of Pharmaceutical Sciences, Uttaranchal University, Dehradun, India; 5 NICM Health Research Institute & School of Science, Western Sydney University, Westmead, NSW 2145 Australia; 6 Department of Biotechnology, Jaypee Institute of Information Technology (JIIT), Noida, Uttar Pradesh India; 7 School of Life Sciences, University of Technology Sydney, Ultimo, NSW 2007 Australia; 8 Department of Translational Research, Dasman Diabetes Institute, Dasman 15462, Kuwait City, Kuwait; 9 Universitas Nusa Cendana, Department of Pharmacy Kupang, Kota Kupang, Nusa Tenggara Timur 85228 Indonesia; 10 Department of Pharmacology and Experimental Therapeutics, College of Pharmacy and Pharmaceutical Sciences, University of Toledo, Toledo, OH 43614 USA; 11 School of Pharmaceutical Sciences, Lovely Professional University, Jalandhar-Delhi GT Road, Phagwara, 144411 Punjab India

**Keywords:** Fisetin, Phytoceuticals, Nanoemulsion, Fecal microbriome extract, Non-small cell lung cancer

## Abstract

Lung cancer (LC) remains the leading cause of cancer-related deaths worldwide, with non-small cell lung cancer (NSCLC) accounting for the majority of cases. While conventional treatments such as chemotherapy and radiotherapy are highly established, adverse effects and therapy resistance necessitate the exploration of novel therapeutic strategies. This study investigates the in vitro anti-cancer potential of the flavonoid fisetin and its nanoemulsion formulation (FNE) in NSCLC, alongside fecal microbiome extract (FME) as a potential adjunct therapy. In vitro functional assays demonstrated that while pure fisetin powder did not significantly inhibit NSCLC cell proliferation, colony formation nor cell migration, FNE and FME exhibited notable cytotoxic effects. Protein array analysis identified significant downregulation of 22 key oncogenic proteins implicated in lung cancer tumour development, epithelial-mesenchymal transition, tumour microenvironment, immunomodulation, therapy resistance, and metastasis. Importantly, the computational analysis supported these results by identifying fisetin-associated genes that correspond to the experimentally downregulated proteins. Computational in silico analysis of fisetin-associated genes identified multiple lung cancer targets including IL6, SERPINE1, PDGFA, MMP3, MST1, MET, and ENPP2. These findings highlight the enhanced therapeutic efficacy achieved through a phytoceutical-loaded nanoemulsion and microbiome-modulated anti-cancer mechanisms, with improved outcomes when combining FNE and FME treatments. The integration of phytoceuticals and microbiome research represents a promising avenue for advancing NSCLC treatment strategies and overcoming current therapeutic limitations.

**Graphical Abstract:**

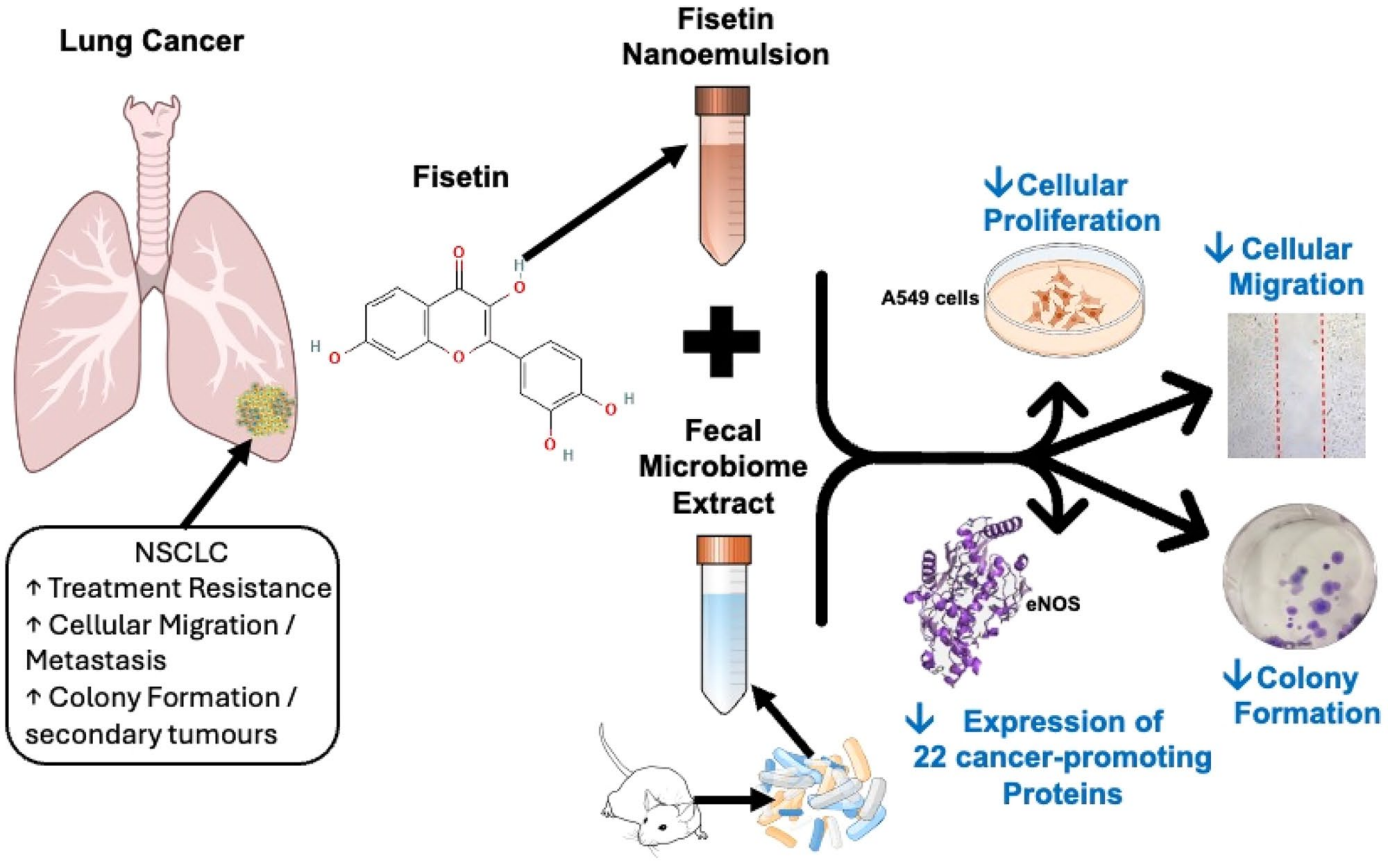

## Introduction

As the leading cause of deaths by cancer worldwide, lung cancer (LC) accounts for approximately 1.8 millions deaths per year [1]. Risk factors for LC development include smoking tobacco cigarettes, use of e-cigarettes and vaping, increased age, environmental pollution, and a family history of the disease [2, 3]. The majority of LC cases (85%) are categorised as non-small cell lung cancer (NSCLC) [1], which has a 5-year survival rate of under 20% [4]. Current standard therapies such as surgery, chemotherapy, and radiation frequently result in adverse effects which burden the patient physically, emotionally, and financially [5]. This leads to a lowered quality of life [6, 7], highlighting the need for complementary and adjunct therapies to improve LC patient wellbeing and improve treatment outcomes. One such field which is gaining increasing momentum is that of phytoceuticals, bioactive compounds sourced from plants [8–10]. Phytoceuticals such as berberine, emodin, quercetin, oridonin, hydroxysafflor yellow A, and ginsenosides have all been shown in vitro to be effective in upregulating anti-oncogenic pathways such as the tumour suppressor, tumour protein 53 (p53), and downregulating pro-oncogenic pathways, including mitogen activated protein kinase (MAPK), keratin 18 (KRT18), and nuclear factor kappa-light-chain-enhancer of activated B cells (NF-κB) [11, 12]. These pathways are involved in many aspects of cancer including oncogenesis, angiogenesis, colony formation and metastasis [11, 12]. This emphasises the potential for, and warrants further investigation of, phytoceuticals and their mechanisms to inhibit cancer formation and progression.

One such phytoceutical with promising therapeutic activity is the flavonoid fisetin (3,3′,4′,7-tetrahydroxyflavone), predominantly found in strawberries, which has been shown to exhibit multiple benefits against a variety of diseases including Alzheimer’s disease, heart disease, hypercholesterolemia, and polycystic ovary syndrome [13–16]. Previous in vitro studies on the NSCLC cell line A549 have shown fisetin to downregulate expression of cyclin-D, cellular Myc (c-Myc), B cell leukemia-lymphoma-2 (Bcl-2), cyclooxygenase-2 (COX-2), matrix metalloproteinase 2 (MMP-2), matrix metalloproteinase 9 (MMP-9) and cluster of differentiation 44 (CD44), which are implicated in tumour progression and metastasis [17, 18]. In other in vitro studies on A549 cells, fisetin has been shown to upregulate anti-oncogenic proteins and pathways such as cyclin-dependent kinase inhibitor 1 A/1B (CDKI 1 A/B) and cyclin-dependent kinase inhibitor 2D (CDKN2D) which promote cessation of the cancer cell cycle [19] and Caspase-3/9 which promotes cancer cell apoptosis [20, 21].

However, despite these promising anti-cancer properties, fisetin, like many phytochemicals, is limited by low bioavailability due to poor water solubility and high lipophilicity [22], requiring novel strategies to improve absorption, pharmacokinetics, and ultimately therapeutic efficacy [22, 23].

In an effort to overcome the above-mentioned challenges, novel strategies including encapsulating phytoceuticals within advanced delivery systems such as liquid crystalline nanoparticles, polymeric nanoparticles, solid lipid nanoparticles, micelles, and liposomes have been investigated [24, 25]. One promising advanced delivery system for phytoceutical delivery is the nanoemulsion, a kinetically stable colloidal dispersion of oil in water [26]. Nanoemulsions have been used to improve the delivery of a variety of phytoceuticals including pterostilbene [27], gallic acid [28], agarwood oil [29], curcumin [30], and berberine [31]. With small particle size in the nano range, nanoemulsions guarantee an increase in surface area for absorption, which improves bioavailability [32]. A fisetin nanoemulson (FNE) has been previously investigated for its in vitro mechanistic anti-cancer effects, showing that the formulation downregulated the LC-associated proteins Cathepsin-B, galectin-3 and Enolase [33], lending itself as an encouraging candidate for further research.

An additional trending area of interest is the gut microbiome, which has been shown to influence multiple systems and pathways including inflammation, allergies, respiratory immune responses [34–36], neurological conditions, behaviour [37, 38], eye diseases including glaucoma [39], and also possess anti-cancer effects [40–42]. Investigating the mechanisms of the microbiome and its metabolites in vitro can be achieved by utilising harvested fecal microbiome extract (FME), as it has been shown that various animal models, such as rats, can mimic various phenotypes of the human intestinal flora [43]. To illustrate the potential of the FME treatment, De Rubis et al. (2024) investigated in vitro the effects of FME on A549 cells. The authors successfully identified the downregulation of six key proteins associated with airway remodelling and lung cancer pathogenesis [44]. Their results confirmed the in vitro potential of FME as treatment for testing mechanistic properties against lung diseases such as Chronic Obstructive Pulmonary Disease (COPD) and NSCLC.

A further strategy to enhance bioactivity of drugs and phytoceuticals is to take advantage of any combined effect between the individual compounds [45]. Recently, in vitro and in vivo studies have identified synergistic effects between phytoceuticals and chemotherapy drugs [45–47]. To complement these in vitro investigations, we also incorporated an in silico analysis to identify fisetin-related gene networks that align with the molecular changes observed experimentally. To complement these in vitro investigations, we also incorporated an in silico analysis to identify fisetin-related gene networks that align with the molecular changes observed experimentally.

The present study shows that FP exerts limited cytotoxicity when applied to A549 cells, with non-significant effect on colony formation and cellular migration. However, utilising a fisetin loaded nanoemulsion (FNE) greatly improves its efficacy, highlighting the benefits of employing an advanced delivery system with poorly soluble bioactive molecules. Additionally, when FNE is combined with a secondary treatment, in this case FME, cytotoxicity is greatly enhanced with superior inhibition of colony formation and cellular migration.

Furthermore, the present study revealed a greater-than-individual response of FNE and FME on the downregulation of all analysed cancer hallmarks. Protein-protein interaction analysis identified upregulation of IL6, SERPINE1, PDGFA, MET, eNOS and AXL which are critical to oncogenic pathways associated with angiogenesis, EMT, proliferation and immune modulation. Additionally, functional enrichment analysis identified a range of biological processes and signalling pathways significantly associated with the target gene set. These included NF-κB signalling, EGFR and VEGFR pathways, PI3K-Akt, JAK-STAT, apoptosis, extracellular matrix disassembly, and cell migration, all of which are integral to lung cancer progression and metastatic potential.

The results highlight the therapeutic potential of combining fisetin-loaded nanoemulsions with fecal microbiome extracts to target key hallmarks of lung cancer progression, offering a novel and integrative strategy within the evolving landscape of cancer treatment focused on natural compounds, nanomedicine, and microbiome modulation. Despite these promising findings, further testing in three-dimensional tumour models and in vivo systems is required to validate efficacy and clarify the specific contributions of microbial species and metabolites within the complex microbiome extract.

## Materials & Methods

### Preparation of Fisetin Powder, Fisetin Nanoemulsion, and Fecal Microbiome Extract

Fisetin (Merck, Australia) was prepared at a concentration of 10 ug/mL in Dulbecco’s Modified Eagle Medium (DMEM, Merck, Australia) supplemented with 5% fetal bovine serum (FBS, Merck, Australia) and 1% Penicillin/Streptomycin (Merck, Australia).

The preparation of FNE has been described in detail in previous reports with optimisation, characterisation, droplet size, PDI, zeta potential and cytotoxicity has been discussed by Kumar et al. [48]. Briefly, a self-nanoemulsifying drug delivery system (SNEDDS) comprising of Castor oil (0.1mL), Lauroglycol FCC (.01mL), Tween (Polysorbate) 80 (0.4mL), Transcutol P (0.6mL), and Fisetin (5 mg) was prepared [33, 48]. The droplet size of the optimised SNEDDS was measured as 154 nm while the zeta potential was − 37 mV. The prepared emulsions were initially assessed by visual inspection to evaluate their spontaneous emulsification behaviour and relative clarity. They were then monitored for up to 48 h to detect any signs of phase separation, creaming, or drug precipitation, thereby confirming their physical stability. Analysis of a placebo FS-SNEDDS showed no interfering signals or unexpected reactivity, confirming any cytotoxic effects were due to fisetin alone and not any of the excipients [48]. The excipients used in our SNEDDS are widely employed pharmaceutical excipients, and multiple in vitro and non-clinical studies have shown low cytotoxicity at concentrations comparable to, or higher than, those present in our formulation [49–52]. The resulting FNE was diluted at a concentration of 10 ug/mL in the supplemented DMEM.

Preparation and compositional profiling of the rat fecal microbiome extract (FME) has been described in previous published reports [41, 44] with characterisation and five main genera identified (Bacteroides(24% ± 2.56%), Cornybacterium (18.1% ± 4.63%), Psychobacter (16.3%, ± 3.1%), Lysinibacillus (11.3% ± 2.32%), and Actinetobacter (9.31% ±2.56%) by Corrie et al. (2022) [41]. Briefly, the FME was prepared from rat fecal material by homogenization in sterile water, followed by centrifugation to remove solids, collection of the clarified supernatant, and lyophilization. During all preparation steps, sterile techniques were maintained to minimize contamination risk. In the present study, all cell culture experiments were performed under standard aseptic conditions using Dulbecco’s Modified Eagle’s Medium (DMEM) supplemented with 1% Penicillin–Streptomycin. Routine visual inspection of culture flasks revealed no turbidity or signs of microbial contamination throughout the experimental period, indicating that the FME preparation did not introduce any viable contaminants. The animal study protocol was approved by Lovely Professional University’s institutional animal ethical committee (Protocol # LPU/IAEC/2021/86). In these experiments, the lyophilized FME aqueous extract was used at a concentration of 10 µg/mL in the supplemented DMEM.

### Cell Culture and Treatment

Human non-small cell lung adenocarcinoma A549 cells were cultured in vitro in DMEM, that had been supplemented with 5% FBS and 1% Penicillin/Streptomycin. The cells were cultured in a humidified incubator with 5% CO2 at 37%. The medium was changed every 48 h until the cells reached 80% confluency. Treatment in all studies was performed for 24 h prior to analysis and data collection.

### MTT Colorimetric Assay

A549 cells were seeded into three 96 well plates at a density of 5,000 cells per well in warmed supplemented DMEM. To allow the cells to adhere to the well surface, the plates were returned to the incubator overnight.

Stock solutions of each treatment (fisetin, FNE, FME and FNE/FME mix) were made at concentrations of 0 (control), 2.5, 5, 10 and 20 µg/mL in DMEM. The spent DMEM in the wells was aspirated and the treatment solutions were added to the wells in 6 replicates per group. The plates were then returned to the incubator for 24 h.

The next day, 3-(4, 5-dimethylthiazolyl-2)-2, 5-diphenyltetrazolium bromide (MTT, Merck, Australia) was added at a concentration of 0.5 mg/mL to each well and left to incubate for 4 h to allow for viable cells which are adhered to the well surface to be stained. After four hours, the supernatant was aspirated from each well and replaced with 100 µl of dimethylsulfoxide (DMSO, Merck, Australia) allowing for the produced formazan to be dissolved into a purple solution. The plates were then analysed using a microplate reader (TECAN Infinite M1000) to assess absorbance at a wavelength of 570 nm to quantify the gradient of colour staining, an indication of cell viability and hence treatment cytotoxicity. Data was analysed using Graphpad PRISM software v10.2.3 (Graphpad Software, Boston, MA, USA), by One-way ANOVA statistical analysis.

### Colony Formation Assay

A549 cells were seeded into three 6 well plates at a concentration of 500 cells per well in warmed supplemented DMEM and allowed to adhere in an incubator overnight.

Successively, the indicated solutions of each treatment were prepared in supplemented DMEM. The supernatant was aspirated from each well. One well each was treated with 2mL of the prepared stock solutions of control (DMEM only, no treatment), Fisetin, FNE, FME, FNE/FME Mix at concentrations of 10 µg/mL, and returned to the incubator for 24 h, after which the medium was refreshed with new supplemented DMEM every 72 h until visible colonies of cells in the control well, approximately after 12 days.

To stain the colonies, the cells were then washed in triplicate with phosphate-buffered saline (PBS, Merck, Australia) followed by fixation with a solution of 4% formaldehyde for 30 min. This was followed by a further triplicate wash with PBS. Crystal violet (Merck, Australia) was then applied at 0.4% w/v, followed by a final triplicate PBS wash. The stained colonies were then allowed to dry overnight before photographic records were taken. Manual counting of colonies was performed.

### Scratch-Wound Assay

The anti-migratory effect of the various fisetin formulations was assessed using a scratch wound assay. A549 cells were cultivated in vitro and then seeded into three 6 well plates at a concentration of 200,000 cells per well. After overnight incubation, a fully confluent monolayer was confirmed in each well.

Solutions of each treatment were made in supplemented DMEM. The supernatant was aspirated from each well. One well each was treated with 2mL of the prepared solutions of control (DMEM only, no treatment), Fisetin, FNE, FME and FNE/FME Mix at concentrations of 10 µg/mL.

Using a sterile 200 µL pipette tip, a single scratch was created in each well. Each scratched well was then photographed noting the time as 0 h, and returned to the incubator for 24 h. Each scratched well was then photographed again, after 24 h. The images were then uploaded to Image J v1.54 g software (Wayne Rasband and contributors, National Institutes of Health, Bethesda, MD, USA) and the distance between the edges of each scratch was measured, with resulting data exported to PRISM Graphpad for statistical analysis.

### Human Oncology Protein Array

The manufacturer’s standard operating procedures and protocols were followed in using the Proteome Profiler Human XL Oncology Array (R&D Systems, Minneapolis, USA) to assess the expression of proteins associated with oncogenesis, cell proliferation and tumour growth, cell migration and metastasis, and chemoresistance.

A549 cells were seeded at a density of 100,000 cells/well/2mL in 6 well plates and incubated overnight to allow for attachment to the well. The cells were then treated with either, FNE, FME or FNE/FME mix at concentrations of 10 µg/mL, with one well untreated as control and incubated for a further 24 h, as indicated above. After treatment, the cells were washed three times with ice-cold PBS. After this, 300µL of radioimmunoprecipitation assay (RIPA) buffer (ThermoFisher Scientific, Australia) supplemented with protease inhibitor cocktail (Roche Diagnostics, Basel, Switzerland) were applied to each well. The cell monolayer was scraped and transferred to a 1.5 mL tube, followed by 30 min incubation on ice and centrifugation at 14,000 g for 15 min at 4 °C to remove cell debris. Protein concentration in the obtained cleared lysate was determined using the bicinchoninic A/acid method (Pierce BCA kit, ThermoFisher Scientific). The cell lysates (300 µg protein/sample) were hybridized on each membrane, and the arrays were developed following the manufacturer’s instructions. The chemiluminescent signal was acquired using a ChemiDoc MP imaging system (Bio-Rad, Hercules, USA). The pixel density of each spot was measured using Image J v1.54 g software (Wayne Rasband and contributors, National Institutes of Health, Bethesda, MD, USA) with resulting data exported for analysis.

### Computational Analysis of Fisetin-Associated Genes in Lung Cancer

#### Gene Selection and Functional Targeting

A curated list of fisetin-associated genes was generated using prior literature, established biological targets and in silico screening tools. Genes were included based on their known involvement in key processes relevant to lung cancer biology, including inflammation, cell proliferation, apoptosis, epithelial–mesenchymal transition and metastasis. This approach ensured that the selected gene set captured pathways central to NSCLC progression and aligned with the protein groups evaluated in our in vitro analyses.

#### Subtype-Specific Expression Analysis Using GSCALite

To explore the relevance of Fisetin-targeted genes across different cancer types, particularly lung adenocarcinoma (LUAD), we employed the GSCALite web platform (http://bioinfo.life.hust.edu.cn/web/GSCALite/). This tool allows for visualization of gene expression across TCGA-defined cancer subtypes. Genes were compared between tumour and normal samples, and statistical significance was determined using FDR-adjusted p-values (Benjamini–Hochberg correction). The results were visualized as bubble plots, where circle size represents statistical significance (− log₁₀FDR), and colour intensity corresponds to the expression change magnitude.

#### Protein-Protein Interaction (PPI) Network Construction

Protein interaction mapping for the selected genes was performed using the STRING v11.5 database (https://string-db.org/). The analysis was restricted to high-confidence interactions (combined score > 0.7), and both direct (physical) and indirect (functional) interactions were included. The resulting interaction network was visualized with nodes representing proteins and edges denoting interactions. Clustered gene modules and central hub proteins were identified to highlight key regulators of Fisetin-modulated pathways [53, 54].

#### Functional Enrichment and Pathway Analysis

Pathway enrichment analysis was conducted using the Database for Annotation, Visualization and Integrated Discovery (DAVID) v6.8 (https://david.ncifcrf.gov/). The uploaded gene set was analyzed for enrichment in Gene Ontology (GO) biological processes and KEGG signaling pathways. Terms with p-values < 0.05 (Benjamini corrected) were considered significantly enriched. Results were plotted as a dot chart, with dot size representing gene count and colour indicating p-value.

#### Pathway Activity Profiling

To investigate the functional consequences of gene expression changes, we assessed pathway activation or inhibition using GSCALite’s pathway activity module. This analysis evaluates each gene’s contribution to hallmark cancer signaling pathways. A heatmap was generated showing the percentage of samples in which a gene was involved in pathway activation or inhibition, providing insight into the mechanistic roles of Fisetin-targeted genes in LUAD.

#### Gene Expression Validation Using GEO Dataset

To validate the expression levels of Fisetin-associated genes in lung cancer, we utilized the publicly available microarray dataset GSE19188 retrieved from the Gene Expression Omnibus (GEO) database (https://www.ncbi.nlm.nih.gov/geo/). This dataset comprises transcriptomic profiles of 91 lung tissue samples, including 45 normal (healthy) and 46 non-small cell lung cancer (NSCLC) specimens.

#### Data Processing and Normalization

Raw microarray expression data were downloaded and processed using the GEO2R tool, which implements the limma package from Bioconductor. Background correction and quantile normalization were applied to ensure consistency across arrays. Probes were annotated using the corresponding platform GPL570 (Affymetrix Human Genome U133 Plus 2.0 Array), and duplicate probe sets mapping to the same gene were averaged.

#### Differential Expression Analysis

Differential expression of Fisetin-responsive genes was assessed by comparing NSCLC samples to normal controls using a moderated t-test. Genes with adjusted p-values (Benjamini-Hochberg correction) < 0.05 were considered statistically significant. Log2-transformed expression values were visualized using boxplots for each gene to illustrate expression differences between normal and cancer tissues.

#### Statistical Analysis and Visualization

Data were analysed and graphs created with GraphPad Prism v10.2.3 (GraphPad Software, Boston, MA, USA) and R ggplot2 package. The obtained data were analysed by ordinary one-way ANOVA, and presented as average ± SEM. Post hoc testing was performed as predetermined in Graphpad Prism using Tukey’s multiple comparison test with singled pool variance. A two-tailed of p-value < 0.05 was considered statistically significant for pairwise comparisons. Utilising in silico analysis of biological processes, including protein–protein interactions, allows the identification and visualisation of relationships between proteins, as well as the common pathways shared between diseases and potential therapeutic compounds [55]. Gene ontology and pathway enrichment analyses were conducted using the DAVID bioinformatics tool (https://david.ncifcrf.gov/), incorporating Kyoto Encyclopedia of Genes and Genomes (KEGG) pathway annotations. PPI networks were generated with the GeneMANIA database and visualised using Cytoscape v3.10.3 [56].

## Results

### FNE and FME Inhibit A549 Cell Proliferation

**Fig. 1:**
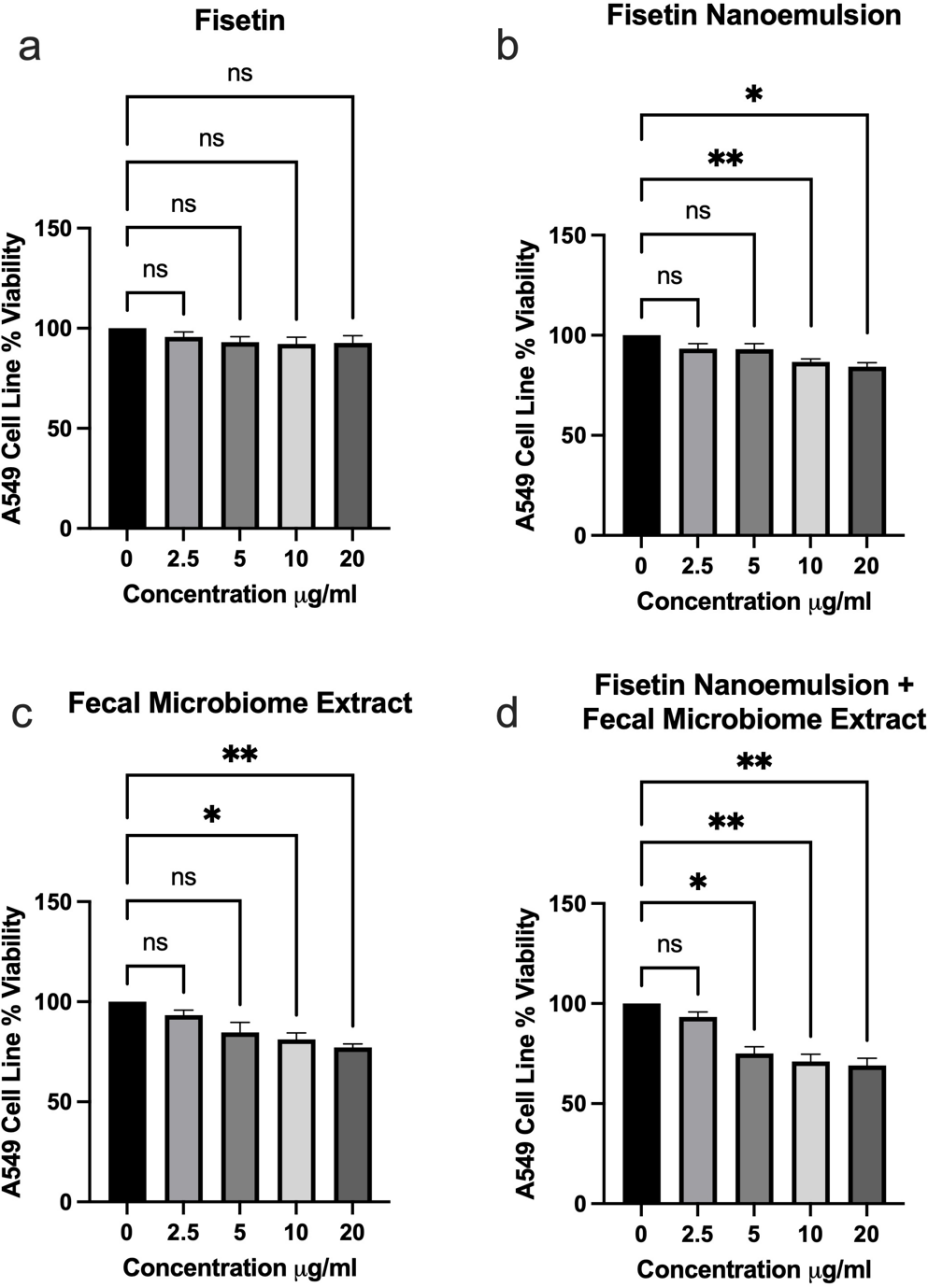
MTT Colorimetric Assay - A549 cells were seeded in 96 well plates at a density of 5000 cells per well. A549 cells were treated with either pure Fisetin Powder (Fig. 1a), Fisetin Nanoemulsion (Fig. 2b), Fecal Microbiome Extract (Fig. 2c) or Fisetin Nanoemulsion / Fecal Microbiome Extract Mix (Fig. 2d) at concentrations of 0, 2.5, 5, 10 and 20 µg/ml and incubated for 24 h. Colorimetric MTT assay was performed to assess cell viability. Data analysed using GraphPad Prism v10.2.3 and One-way ANOVA analysis. Results are expressed as *±* SEM (*n* = 3), *p* < 0.05 (*); *p* < 0.01 (**); ns: not significant

Treatment of A549 cells with up to 20 µg/mL of FP had no significant impact on cell proliferation (Fig. 1a). Treatment with less than 10 µg/mL of either FNE or FME had no significant impact on cell proliferation (Fig. 1b and c, respectively). Treatment with up to 2.5 µg/mL of the combined FNE/ FME Mix had no significant impact on cell proliferation (Fig. 1d). However, when compared to control, treatment with 10 and 20 µg/mL of FNE resulted in a significant 13.49% and 15.65% reduction in cell proliferation, respectively (Fig. 1b). Treatment with 10 and 20 µg/mL of FME resulted in a significant 18.77% and 22.76% reduction in cell proliferation, respectively, compared to the untreated control group (Fig. 1c). Furthermore, treatment with 5, 10 and 20 µg/mL of combined FNE/FME Mix resulted in significant reductions of cell proliferation by 25.05%, 28.92% and 30.98%, respectively, compared to the untreated control group (Fig. 1d).

### FNE and FME Reduce A549 Colony Formation

**Fig. 2:**
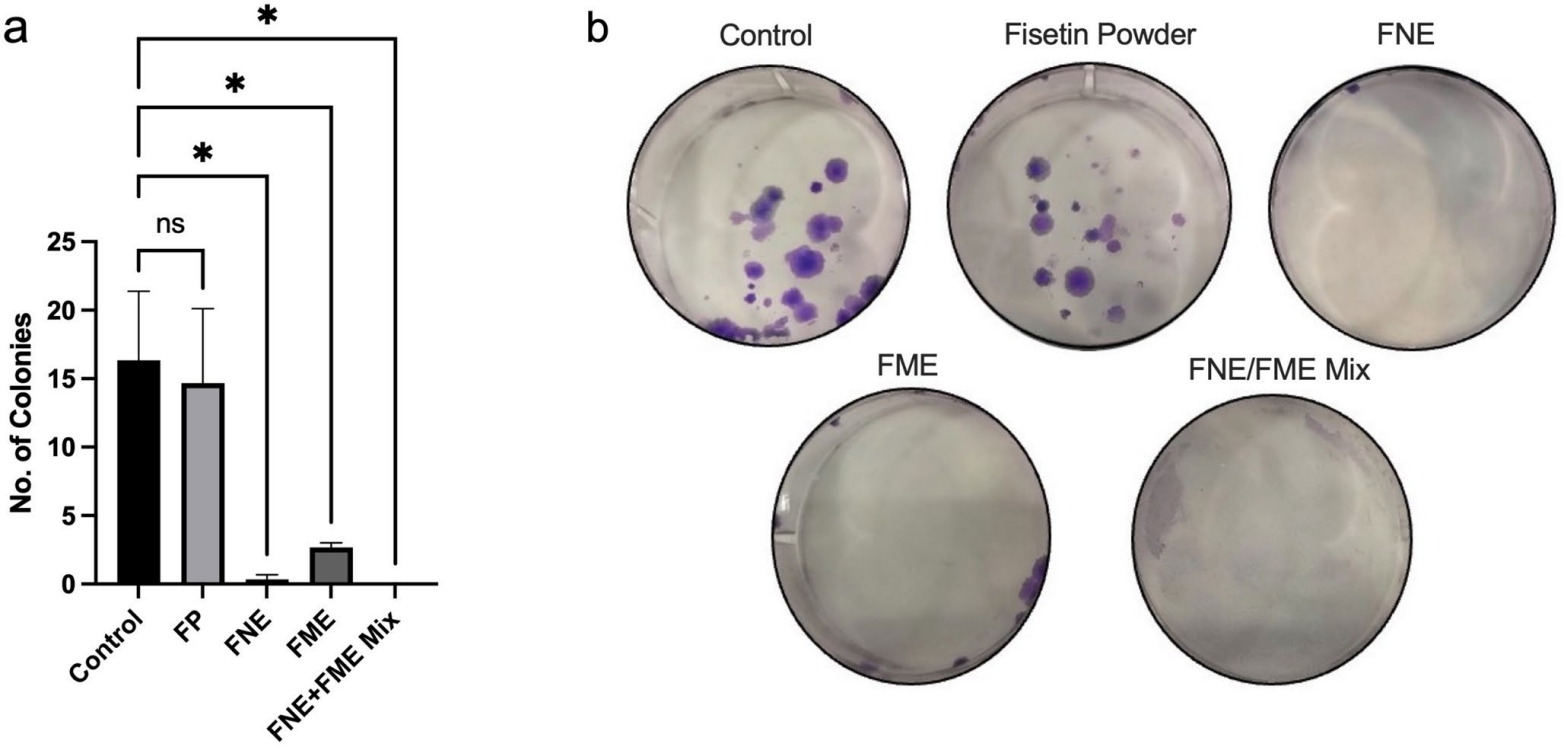
Colony Formation - A549 cells were seeded in 6 well plates at 500 cell per well. Colony formation of A549 cells after treatment of FP, FNE, FME and FNE/FME mix. Each well treated with FP, FNE, FME or FNE/FME Mix at concentrations of 10 µg/mL each, then cultured for 12 days. Colonies were stained with crystal violet, photographed and counted manually. Data analysed using GraphPad Prism v10.2.3 and One-way ANOVA analysis. Results expressed as *±* SEM (*n* = 3), *p* < 0.05(*); ns: not significant. Figure 2b shows representative images from the three independent experiments

The results of the colony formation assay are showed in Fig. 2. The resulting data indicate that, compared to control, FP did not significantly inhibit the capacity of A549 cells to form colonies when seeded at very low density (Fig. 2a). Treatment with FNE and FME significantly inhibited A549 colony formation by 97.96% and 83.67% respectively (Fig. 2a). The FNE/FME mix had the greatest effect with zero colonies formed, that is, a 100% reduction of colony formation when compared to control. Representative pictures of the colonies formed are shown in Fig. 2b.

### FNE and FME Inhibit A549 Cell Migration

**Fig. 3:**
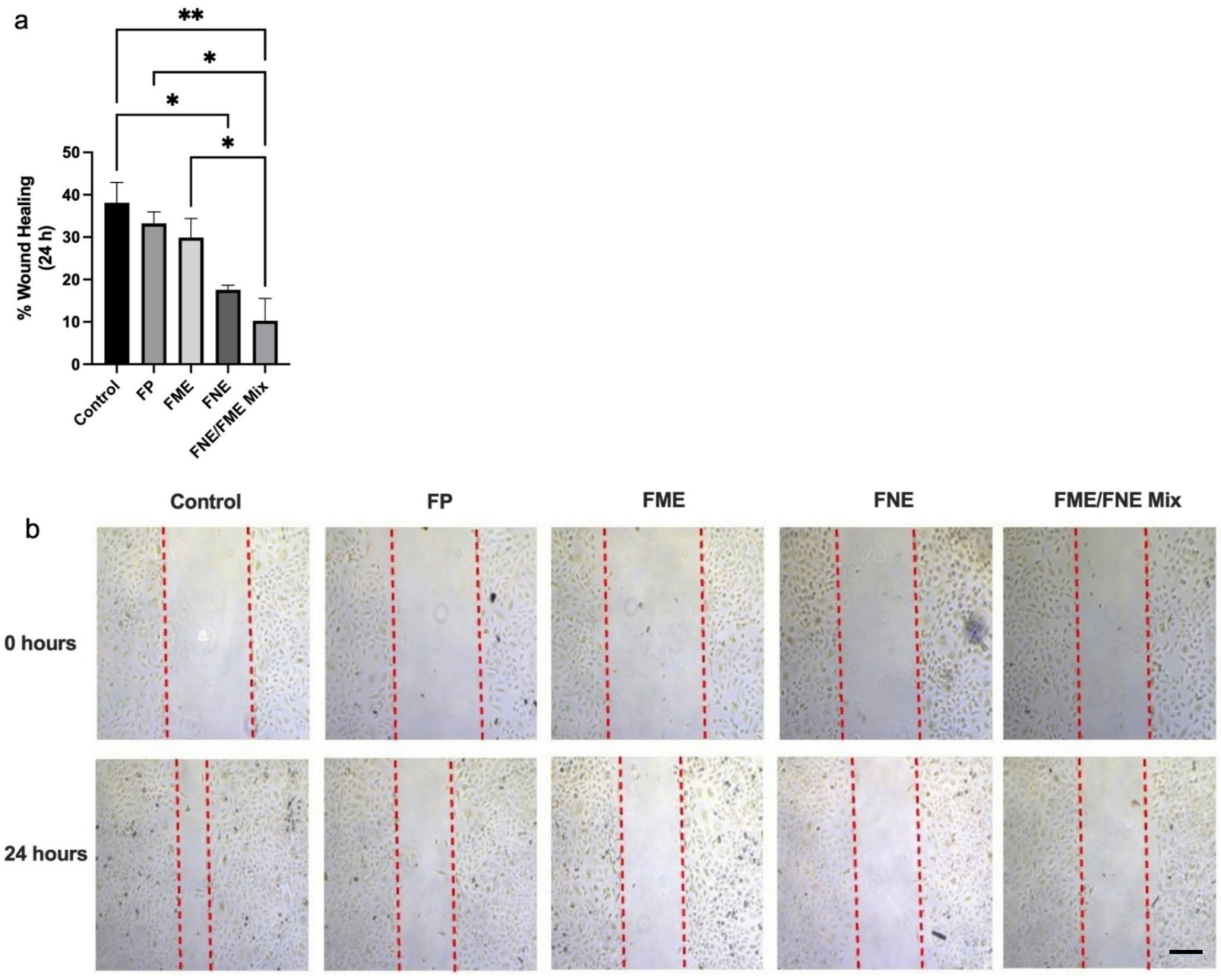
Cell Migration Scratch Wound Assay - A549 cells were seeded into three 6 well plates at a concentration of > 200,000 cells per well. A single scratch was created in each well with a sterile 200 µL pipette tip. Wells were then treated with FP, FNE, FME or FNE/FME Mix, and incubated for 24 h. Photographs were analysed with Image J v1.54 g software Data analysed using GraphPad Prism v10.2.3 and One-way ANOVA analysis. Figure 3b shows representative images from the three independent experiments. Results expressed ± SEM (*n* = 3), *p* < 0.05(*), *p* < 0.01(**). Scale Bar = 300 μm

The results of the scratch-wound assay are depicted in Fig. 3. After 24 h of treatment, the control group had a wound closure of 38.08% (Fig. 3a). Treatment with either FP or FME did not result in a significant inhibition of wound closure when compared to control (Fig. 3a). However, treatment with FNE significantly inhibited wound closure by 53% (Fig. 3a), and the FNE/FME mix significantly inhibited wound closure by 73.04% (Fig. 3a). Treatment with FNE/FME mix resulted in a significant 69.09% and 65.66% inhibition of wound closure when compared to than FP and FME, respectively (Fig. 3a). Representative pictures of the scratch-wound assay are shown in Fig. 3b.

### FNE & FME Downregulates Proteins Associated with Hallmarks of Lung Cancer

We report the significant downregulation of 22 proteins after exposure to FNE, FME, or the FNE/FME mix. These proteins are implicated in various aspects of lung cancer including but not limited to oncogenesis, cell migration and metastasis, colony formation, epithelial-mesenchymal transition, and chemoresistance. These proteins are Amphiregulin, Angiopoietin-like 4, Axl, BCL-x, Carbonic Anhydrase IX, Cathepsin S, CCL8, Enolase, Endothelial nitric oxide synthase (eNOS), ENPP-2/Autotaxin, EpCAM, FoxC2, HCG α/β, HGFR/c-MET, IL-2Ra (CD25), IL-6, Kallikrein-6, MMP-3, MSP/MST1, PDGF-AA, Serpin E1, and Survivin.

### FNE and FME Downregulate the Expression of Proteins Associated with Lung Cancer Invasion and Metastasis

**Fig. 4:**
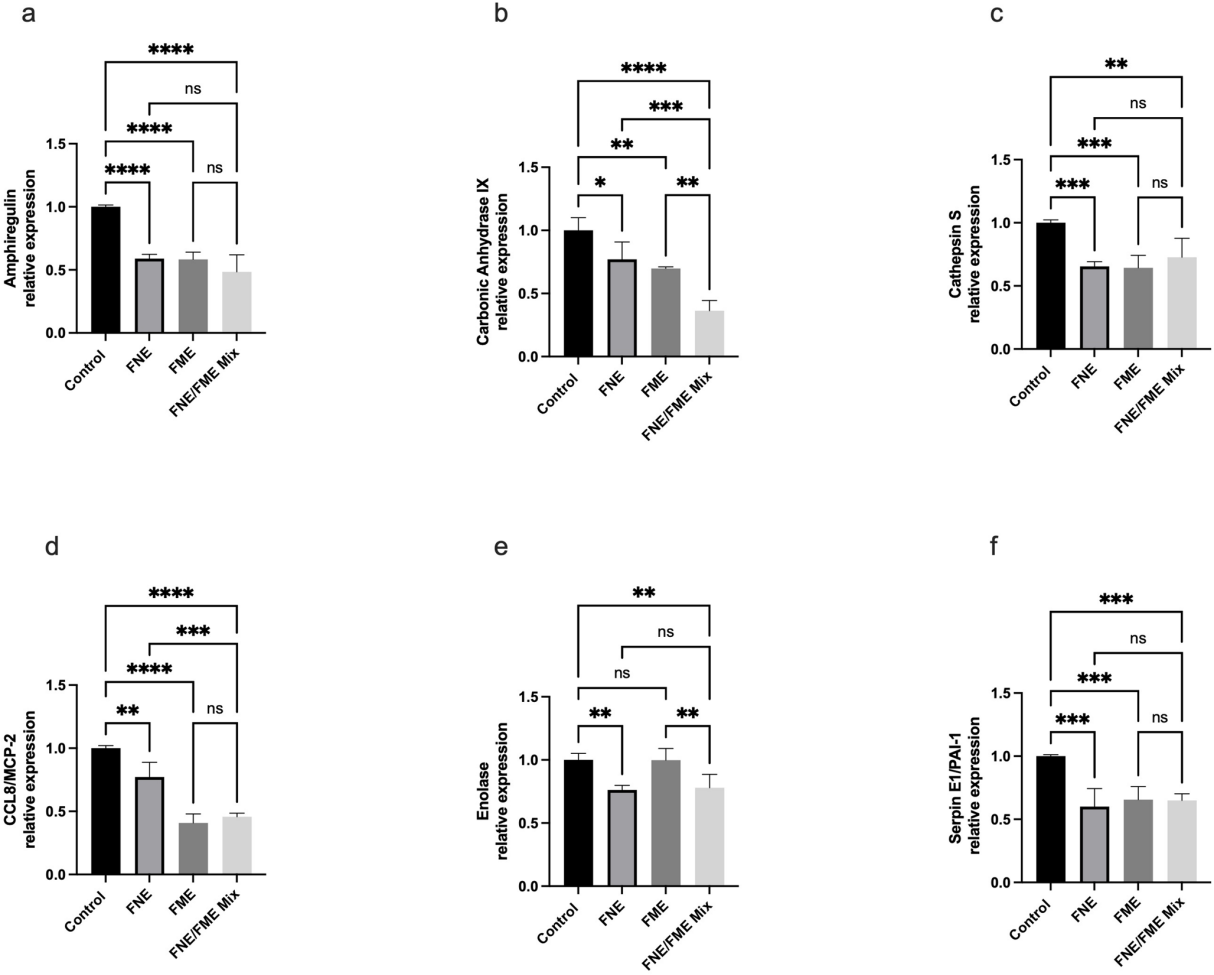
Human Oncology Protein Array - Effect of Fisetin Nanomulsion (FNE), Fecal Microbiome Extract (FME) and FNE/FME Mix on inhibition of proteins associated with lung cancer invasion and metastasis (**a**) Amphiregulin, (**b**) Carbonic Anydrase IX, (**c**) Cathepsin S, (**d**) CCL8, (**e**) Enolase, (**f**) Serpin E1/PAI-1 expression in A549 cells as assessed via protein array; Data analysed using GraphPad Prism v10.2.3 and One-way ANOVA analysis. Results expressed + SEM (*n* = 4); *p* < 0.05(*), *p* < 0.01(**), *p* < 0.001(***); *p* < 0.0001(****); ns: not significant vs. control

Relative to the control, Amphiregulin expression (Fig. 4a) was significantly suppressed following treatment with FNE (41.05% reduction), FME (41.66% reduction), and the FNE/FME mix (51.52% reduction). Relative to control, Carbonic anhydrase IX expression (Fig. 4b) was significantly suppressed following treatment FNE (22.95% reduction), FME (30.23%), and the FNE/FME mix (63.69). Relative to control Cathepsin S expression (Fig. 4c) was significantly expressed following treatment with FNE (34.54%), FME (35.63%,), and the FNE/FME mix (27.28%). Relative to control CCL8 expression (Fig. 4d) was significantly suppressed following treatment with FNE (22.83), FME (59.24%) and the FNE/FME mix (54.35%). Relative to control, Enolase expression (Fig. 4e) was significantly suppressed following treatment with FNE (23.81%) and the FNE/FME mix (22.05%). Relative to control, Serpin E1 expression (Fig. 4f) was significantly suppressed following treatment with FNE (39.90%), FME (34.38%and the FNE/FME mix (35.02%).

### FNE & FME Downregulate the Expression of Proteins Associated with Lung Cancer Tumour Progress, Cell Proliferation and Colony Formation

**Fig. 5:**
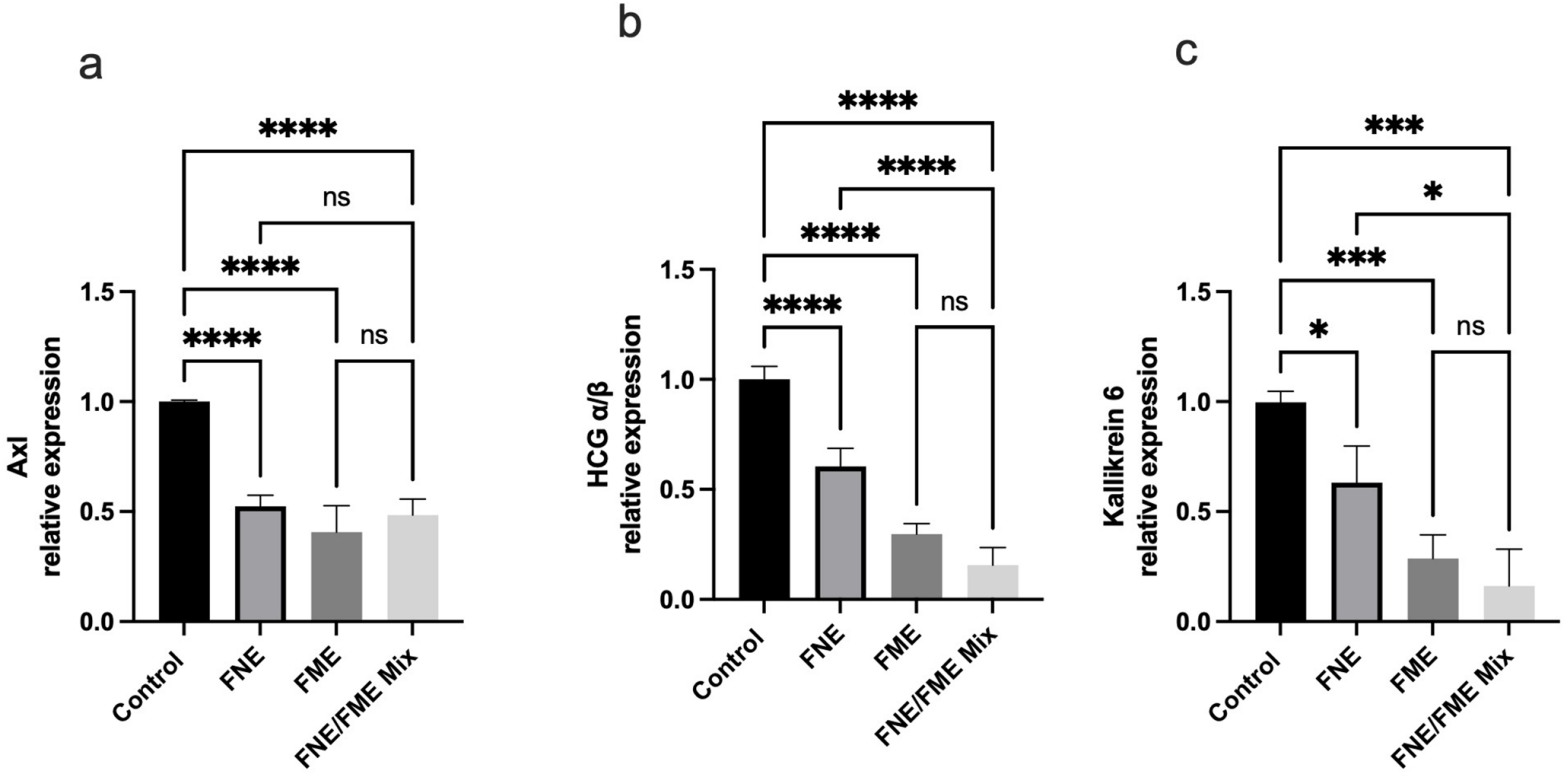
Effect of Fisetin Nanomulsion (FNE), Fecal Microbiome Extract (FME) and FNE/FME Mix on inhibition of proteins associated with lung cancer tumour progress, cell proliferation and colony formation (**a**) Axl, (**b**) HCG α/β, (**c**) Kallikrein-6 expression in A549 cells as assessed via protein array; Data analysed using GraphPad Prism v10.2.3 and One-way ANOVA analysis. Results expressed + SEM (*n* = 4); *p* < 0.05(*), *p* < 0.01(**), *p* < 0.001(***); *p* < 0.0001(****) ns: not significant vs. control

Relative to the control, Axl expression (Fig. 5a) was significantly suppressed following treatment with FNE (47.58% reduction), FME (59.28% reduction, *p* < 0.0001), and the FNE/FME mix (51.51% reduction). Relative to control HCG α/β expression (Fig. 5b) was significantly suppressed following treatment with FNE (39.51%FME (70.31%), and the FNE/FME mix (84.46%). Relative to control Kallikrein-6 expression (Fig. 5c) was significantly suppressed following treatment with FNE (30.73%, *p* < 0.05), FME (68.28%) and the FNE/FME mix (68.28%).

### FNE & FME Downregulate the Expression of Proteins Associated with Lung Cancer Epithelial-Mesenchymal Transition

**Fig. 6:**
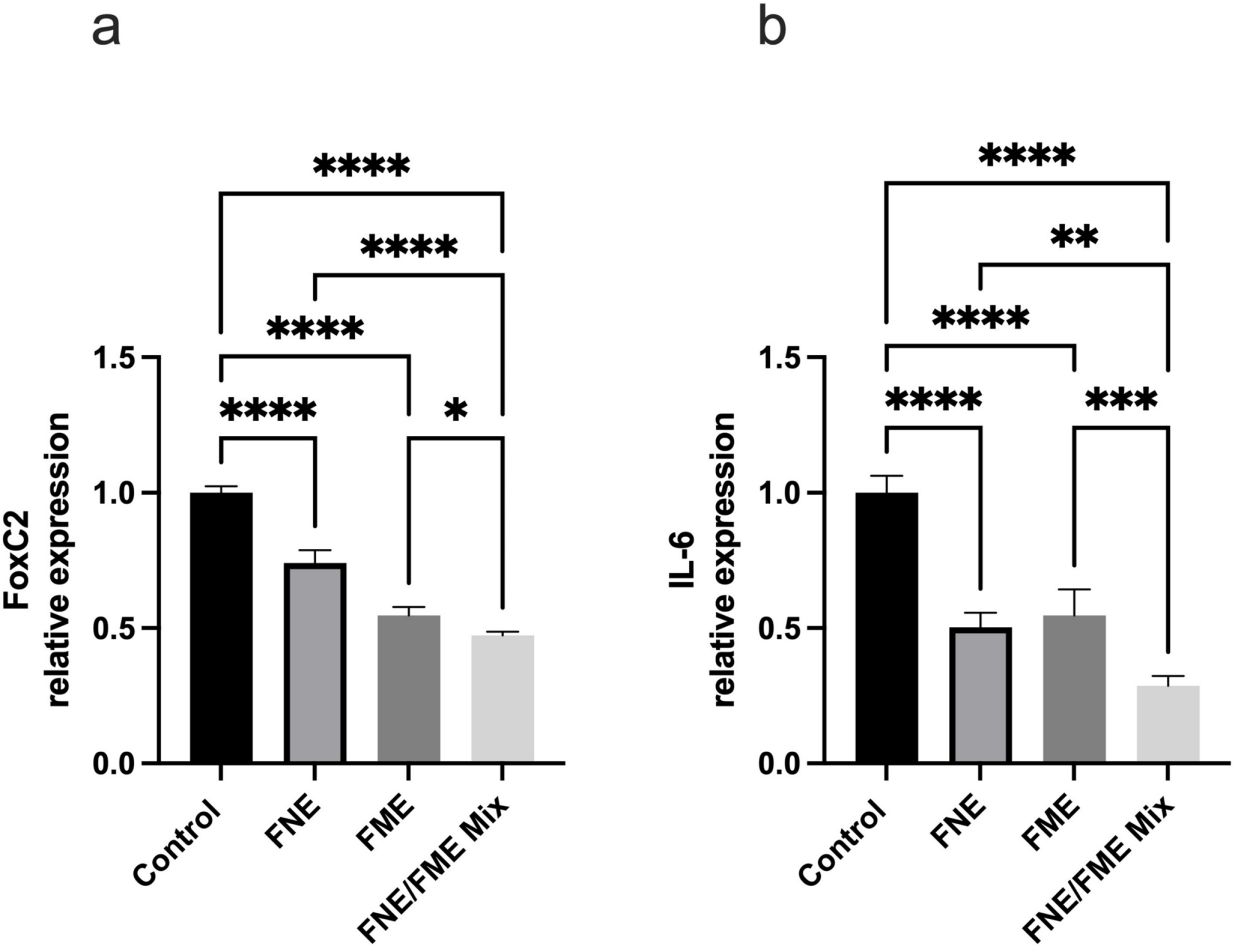
Effect of Fisetin Nanomulsion (FNE), Fecal Microbiome Extract (FME) and FNE/FME Mix on inhibition of proteins associated with lung cancer epithelial-mesenchymal transition (a) FoxC2, (b) IL-6 expression in A549 cells as assessed via protein array; Data analysed using GraphPad Prism v10.2.3 and One-way ANOVA analysis. Results expressed + SEM (*n* = 4); *p* < 0.05(*), *p* < 0.01(**), *p* < 0.001(***); *p* < 0.0001(****) vs. control

Relative to the control, FoxC2 expression (Fig. 6a) was significantly suppressed following treatment with FNE (25.97% reduction), FME (45.32% reduction), and the FNE/FME mix (52.74% reduction). Relative to the control, IL-6 expression (Fig. 6b) was significantly suppressed following treatment with FNE (49.74% reduction), FME (45.33% reduction), and the FNE/FME mix (71.28% reduction).

### FNE & FME Downregulate the Expression of Proteins Associated with Lung Cancer Tumour Angiogenesis

**Fig. 7:**
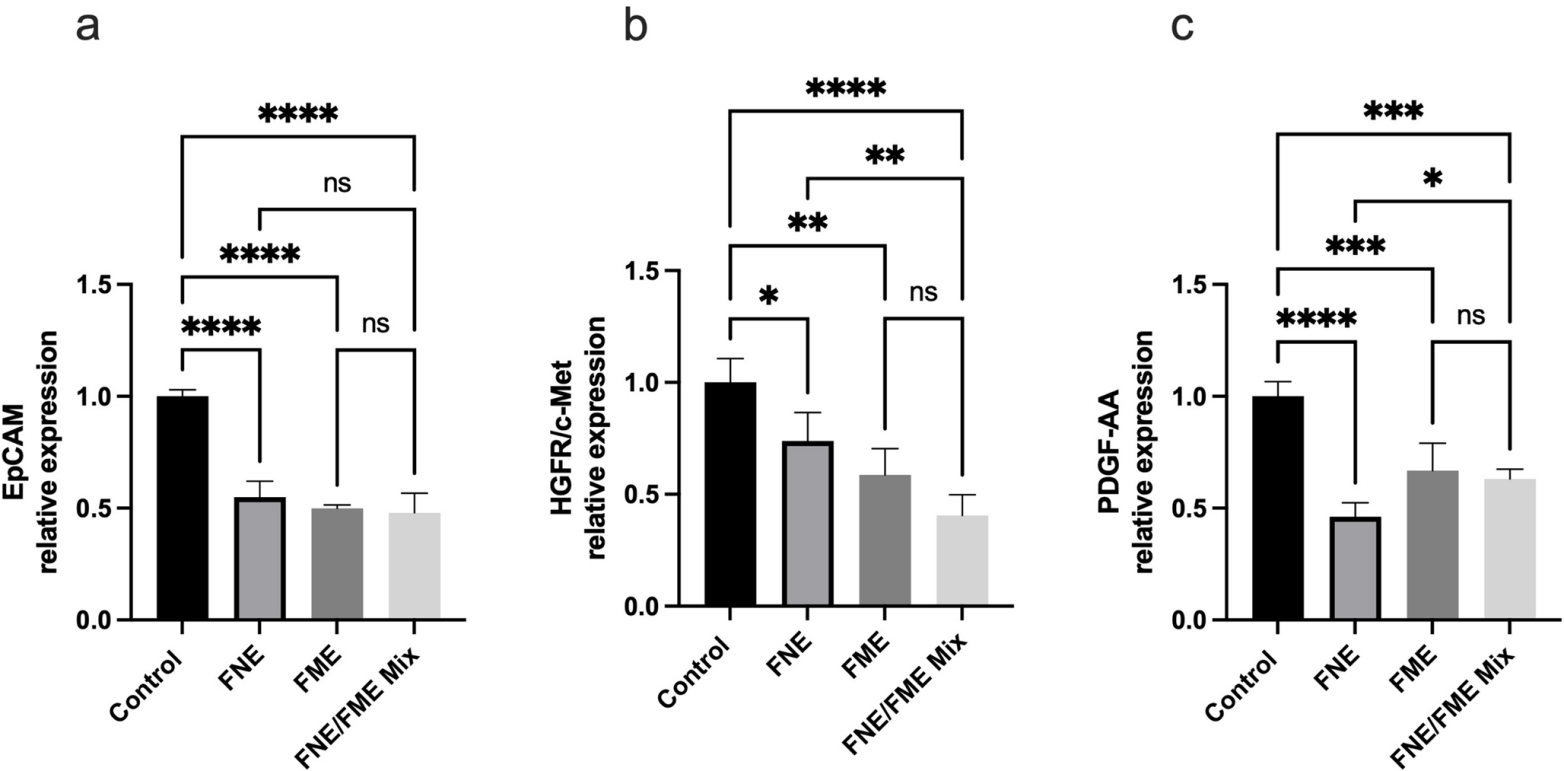
Effect of Fisetin Nanomulsion (FNE), Fecal Microbiome Extract (FME) and FNE/FME Mix on inhibition of proteins associated with lung angiogenesis (**a**) EpCAM, (**b**) HGFR/c-Met, (c) PDGF-AA expression in A549 cells as assessed via protein array; Data analysed using GraphPad Prism v10.2.3 and One-way ANOVA analysis. Results expressed + SEM (*n* = 4); *p* < 0.05(*), *p* < 0.01(**), *p* < 0.001(***); *p* < 0.0001(****) ns: not significant vs. control

Relative to the control, EpCAM expression (Fig. 7a) was significantly suppressed following treatment with FNE (45.00% reduction), FME (50.09% reduction), and the FNE/FME mix (52.03% reduction). Relative to the control, HGFR/c-MET expression (Fig. 7b) was significantly suppressed following treatment with FNE (26.17% reduction), FME (41.29% reduction), and the FNE/FME mix (59.40% reduction). Relative to the control, PDGF-AA expression (Fig. 7c) was significantly suppressed following treatment with FNE (53.80% reduction), FME (33.15% reduction), and the FNE/FME mix (36.93% reduction).

### FNE & FME Downregulate the Expression of Proteins Associated with Lung Cancer Therapy Resistance

**Fig. 8:**
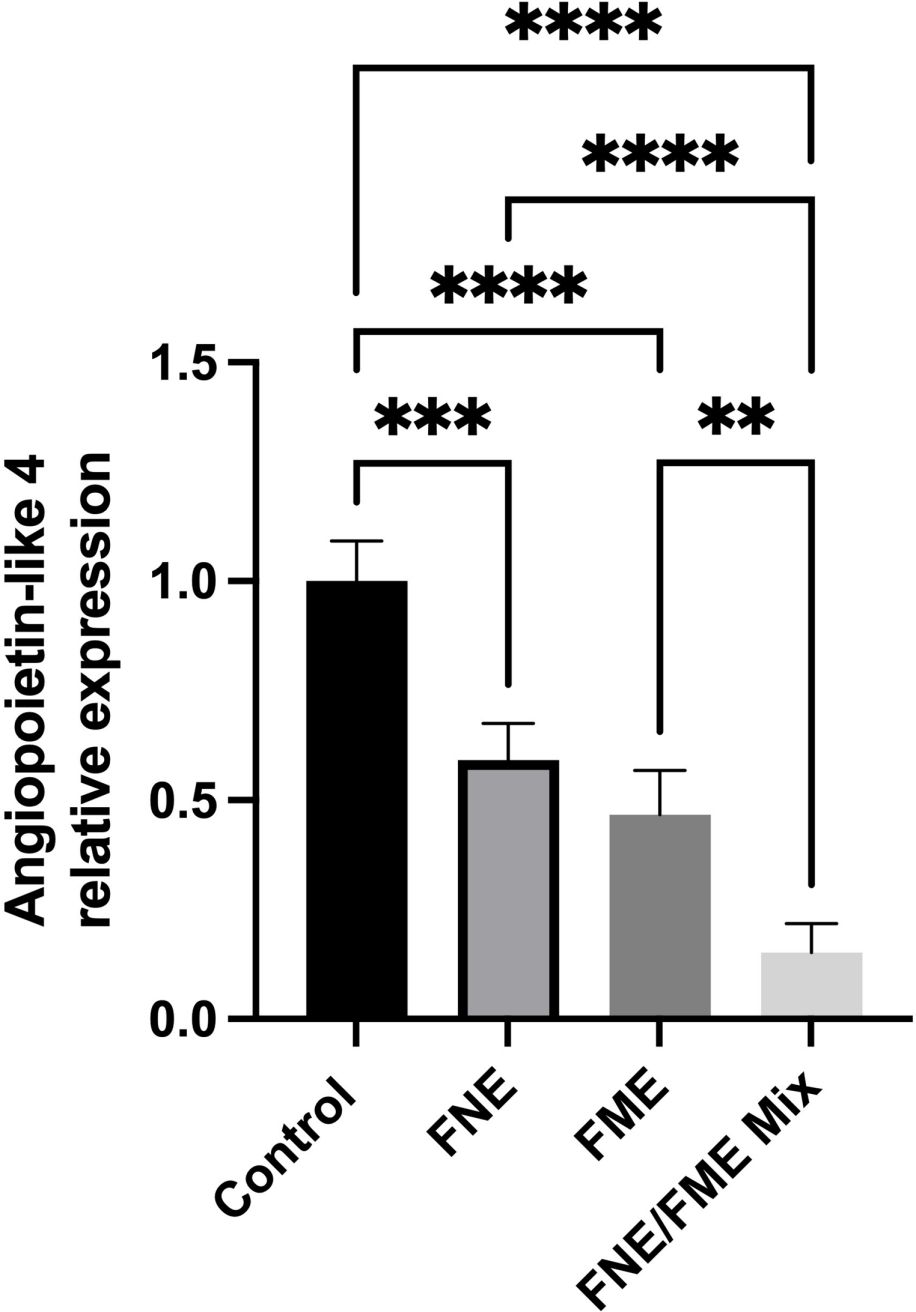
Effect of Fisetin Nanomulsion (FNE), Fecal Microbiome Extract (FME) and FNE/FME Mix on inhibition of proteins associated with lung cancer therapy resistance a) Angiopoietin-like 4 expression in A549 cells as assessed via protein array; Data analysed using GraphPad Prism v10.2.3 and One-way ANOVA analysis. Results expressed + SEM (*n* = 4); *p* < 0.01(**), *p* < 0.001(***); *p* < 0.0001(****) vs. control

Relative to the control, Angiopoietin-like 4 expression (Fig. 8) was significantly suppressed following treatment with FNE (40.80% reduction), FME (53.36% reduction), and the FNE/FME mix (84.79% reduction).

### FNE & FME Downregulate the Expression of Proteins Associated with Lung Cancer Tumour Microenvironment

**Fig. 9:**
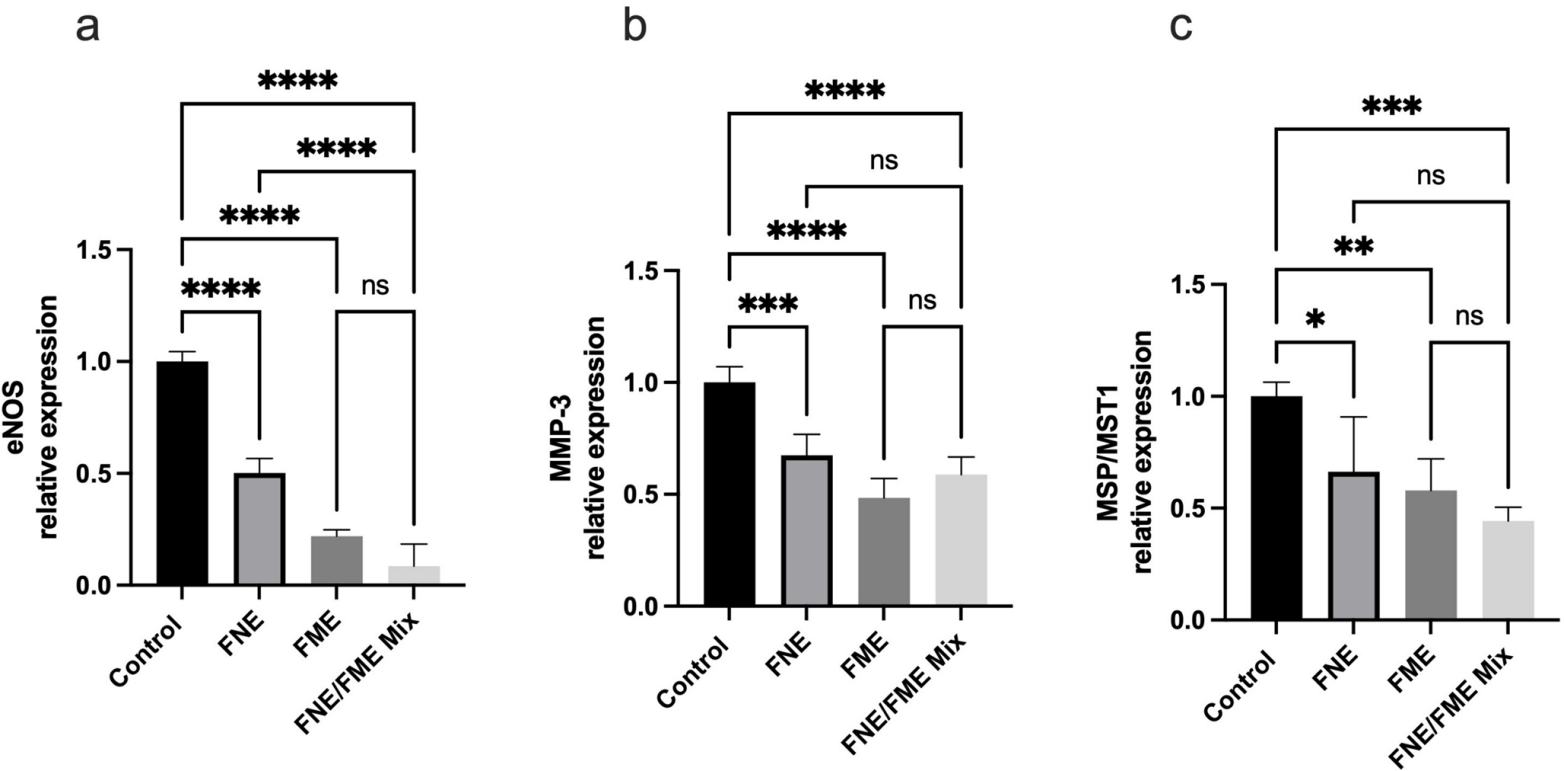
Effect of Fisetin Nanomulsion (FNE), Fecal Microbiome Extract (FME) and FNE/FME Mix on inhibition of proteins associated with lung cancer tumour microenvironment (**a**) eNOS, (**b**) MMP-3, (**c**) MSP/MST1 expression in A549 cells as assessed via protein array Data analysed using GraphPad Prism v10.2.3 and One-way ANOVA analysis. Results expressed + SEM (*n* = 4); *p* < 0.05(*), *p* < 0.01(**), *p* < 0.001(***); *p* < 0.0001(****) ns: not significant vs. control

Relative to the control, eNOS expression (Fig. 9a) was significantly suppressed following treatment with FNE (49.76% reduction), FME (78.12% reduction), and the FNE/FME mix (91.49% reduction). Relative to the control, MMP-3 expression (Fig. 9b) was significantly suppressed following treatment with FNE (32.61% reduction), FME (51.51% reduction), and the FNE/FME mix (41.16% reduction). Relative to control, MSP/MST1 expression (Fig. 9c) was significantly suppressed following treatment with FNE (33.68% reduction), FME (42.10% reduction), and the FNE/FME mix (55.69% reduction).

### FNE & FME Downregulate the Expression of Proteins Associated with Lung Cancer Anti-apoptosis

**Fig. 10:**
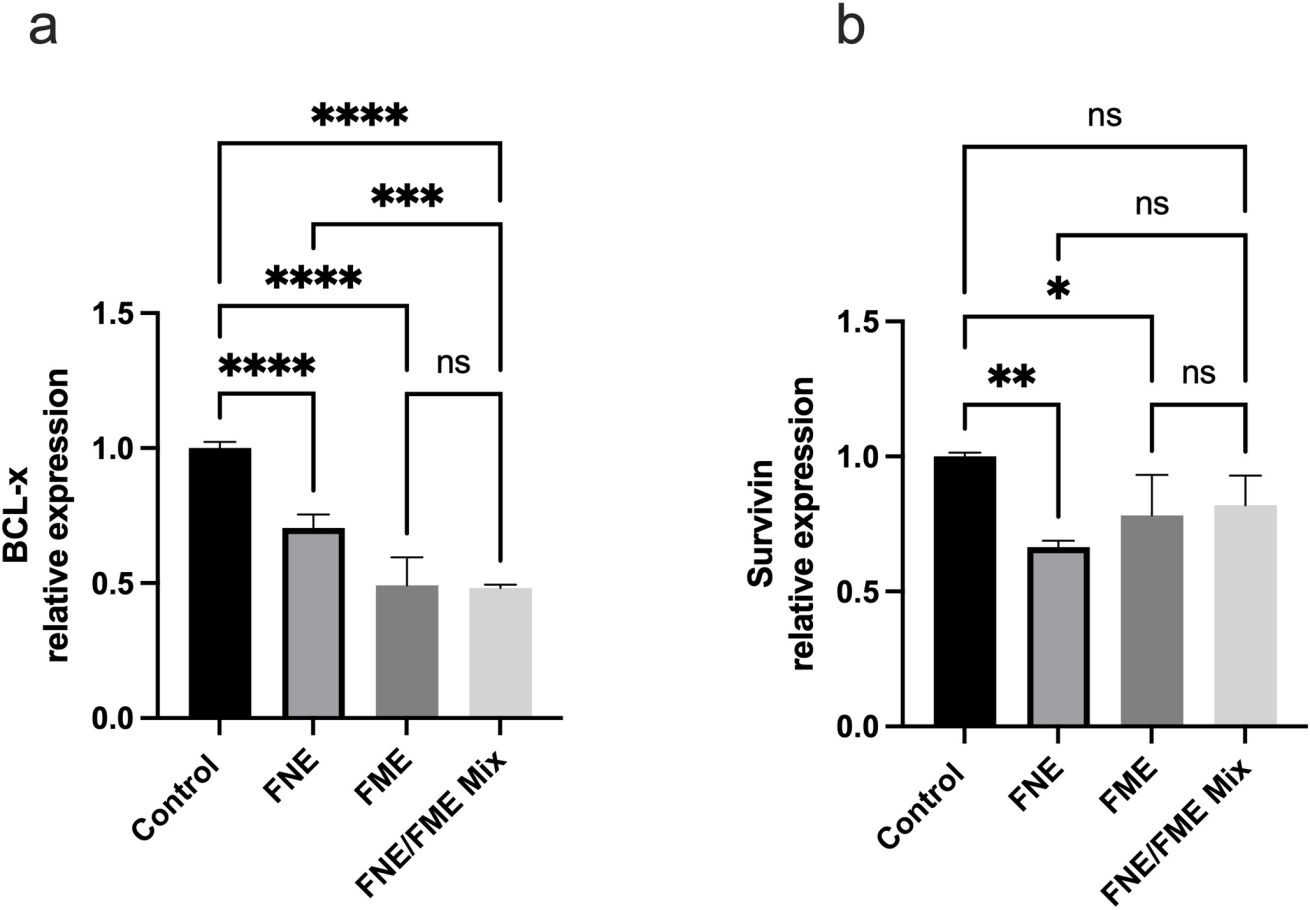
Effect of Fisetin Nanomulsion (FNE), Fecal Microbiome Extract (FME) and FNE/FME Mix on inhibition of proteins associated with lung cancer anti-apoptosis (**a**) BCL-x, (**b**) Survivin expression in A549 cells as assessed via protein array. Data analysed using GraphPad Prism v10.2.3 and One-way ANOVA analysis. Results expressed + SEM (*n* = 4); *p* < 0.05(*), *p* < 0.01(**), *p* < 0.001(***); *p* < 0.0001(****) ns: not significant vs. control

Relative to the control, BCL-x expression (Fig. 10a) was significantly suppressed following treatment with FNE (29.59% reduction), FME (50.79% reduction), and the FNE/FME mix (50.79% reduction). Relative to the control, Survivin expression (Fig. 10b) was significantly suppressed following treatment with FNE (33.54% reduction) and FME (21.87% reduction).

### FNE & FME Downregulate the Expression of Proteins Associated with Lung Cancer Immunomodulation

**Fig. 11:**
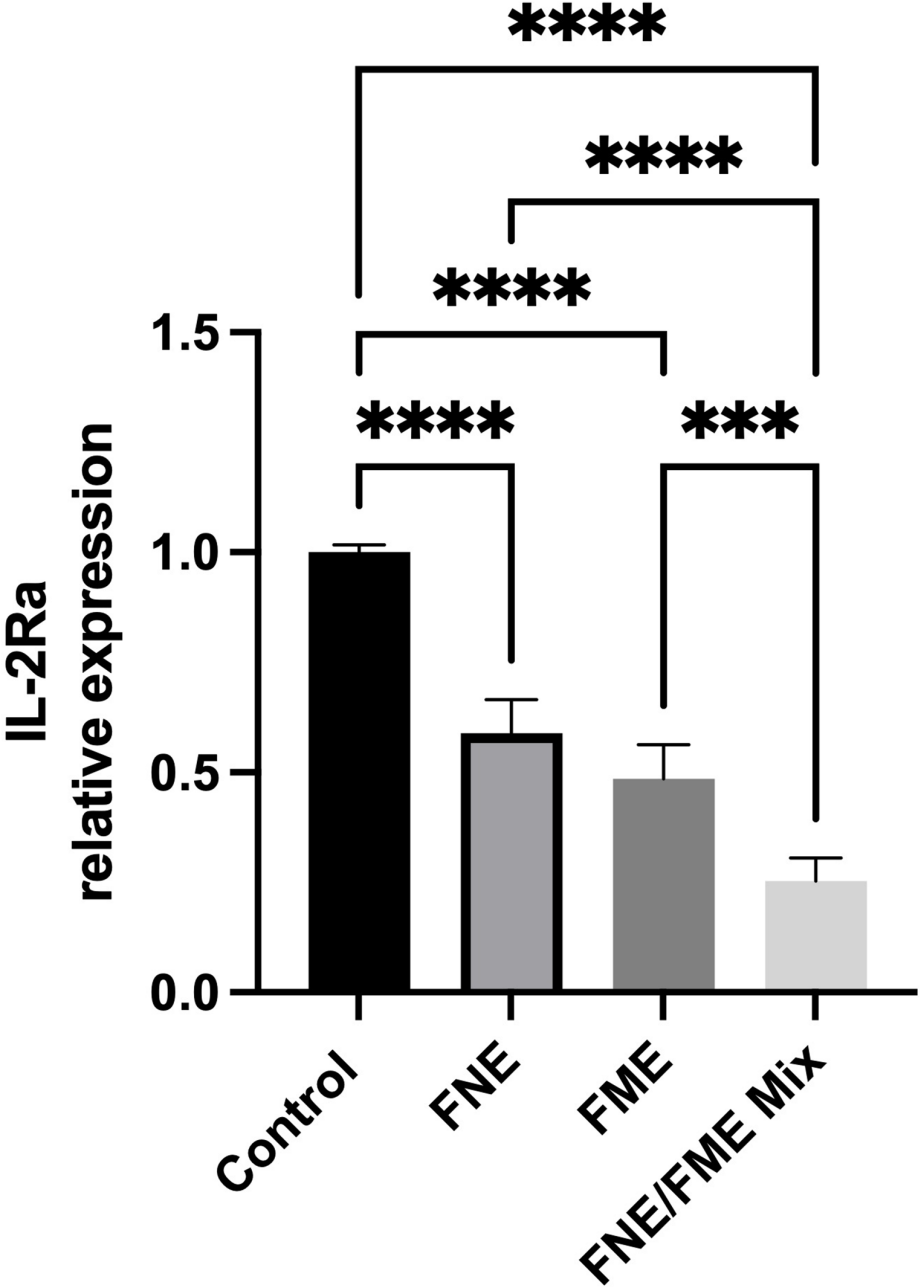
Effect of Fisetin Nanomulsion (FNE), Fecal Microbiome Extract (FME) and FNE/FME Mix on inhibition of proteins associated with lung cancer immunomodulation a) IL-2Ra expression in A549 cells as assessed via protein array; Data analysed using GraphPad Prism v10.2.3 and One-way ANOVA analysis. Results expressed + SEM (*n* = 4); *p* < 0.001(***); *p* < 0.0001(****) vs. control

Relative to the control, IL-2Ra expression was significantly suppressed following treatment with FNE (41.03% reduction), FME (51.43% reduction), and the FNE/FME mix (74.65% reduction) (Fig. 11).

### FNE & FME Downregulate the Expression of Proteins Associated with Lung Cancer Oncogenesis

**Fig. 12:**
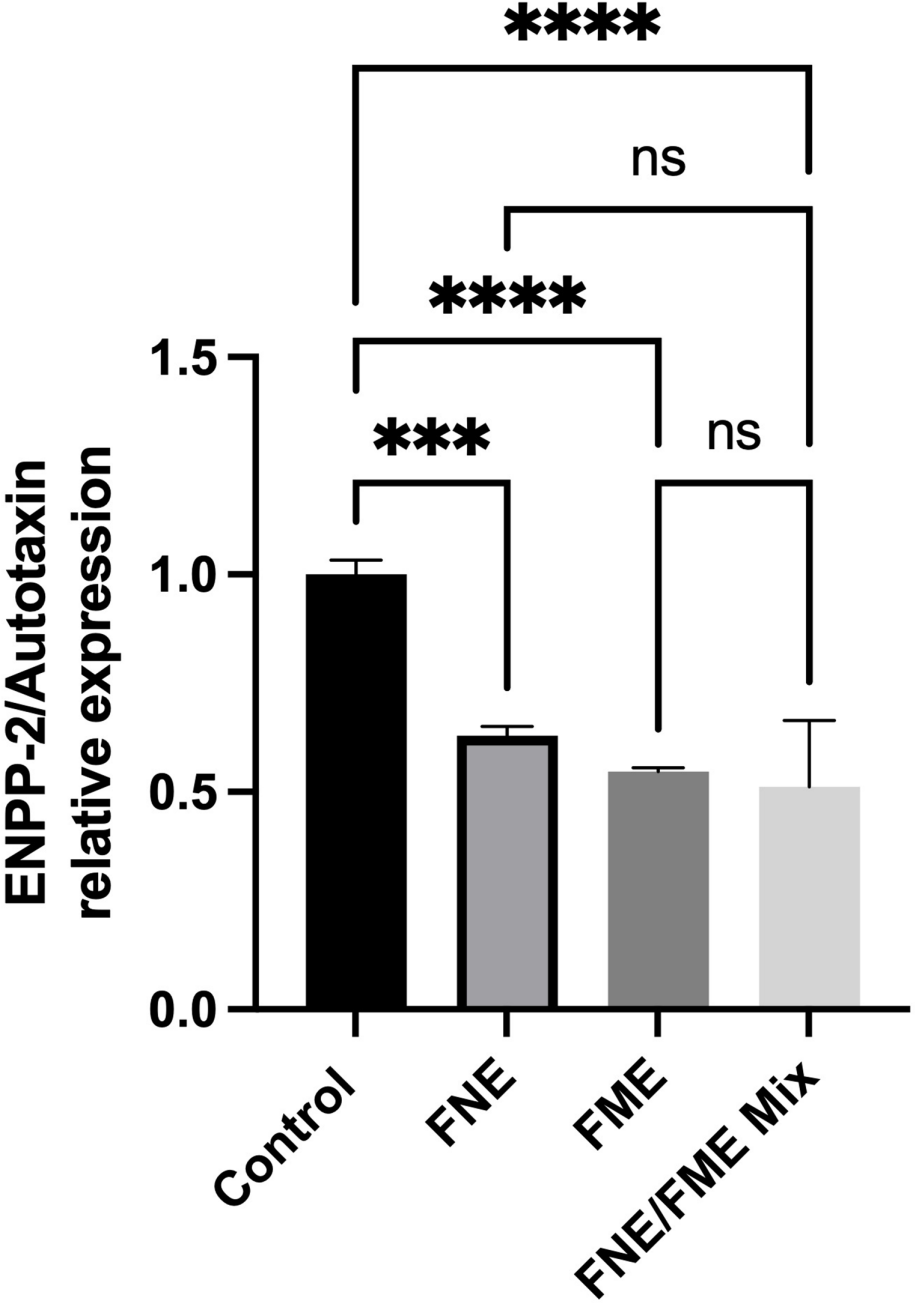
Effect of Fisetin Nanomulsion (FNE), Fecal Microbiome Extract (FME) and FNE/FME Mix on inhibition of proteins associated with lung cancer oncogenesis a) ENPP-2/Autotaxin expression in A549 cells as assessed via protein array; Data analysed using GraphPad Prism v10.2.3 and One-way ANOVA analysis. Results expressed + SEM (*n* = 4); *p* < 0.001(***); *p* < 0.0001(****); ns: not significant vs. control

Relative to the control, ENPP-2/Autotaxin expression was significantly suppressed following treatment with FNE (37.05% reduction), FME (45.29% reduction), and the FNE/FME mix (48.8% reduction). (Fig. 12)

## Computational Network and Pathway Analysis Reveals Dysregulated Oncogenic Signatures of Fisetin-Targeted Genes in Lung Adenocarcinoma

**Fig. 13:**
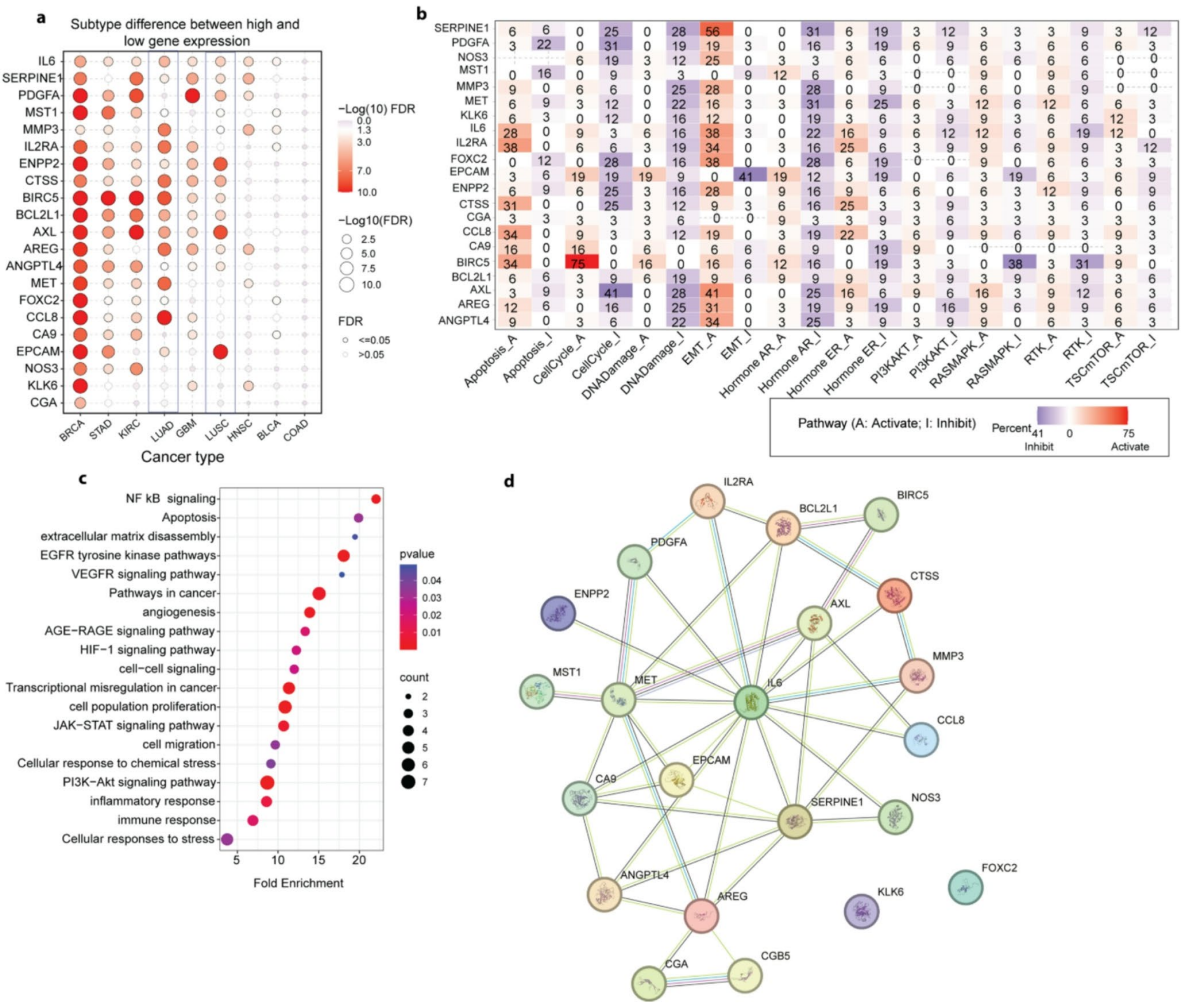
Integrated in silico analysis of fisetin-associated molecular networks in lung adenocarcinoma. (**a**) Bubble plot showing subtype-specific differences in gene expression across various cancer types using the GSCALite tool. Genes targeted by Fisetin were significantly upregulated in LUAD and other cancers. The colour intensity indicates the − log₁₀(FDR) value, and bubble size corresponds to the statistical significance. (**b**) Heatmap of pathway activity from GSCALite showing the regulatory role of each gene across key cancer-related signalling pathways. Red denotes pathway activation, blue indicates inhibition, and white indicates no effect. The percentage reflects how frequently each gene regulates the respective pathway (**c**) Pathway enrichment analysis highlighting the most significantly enriched biological processes relevant to tumour progression, displayed by fold enrichment, gene count and p-value. Dot size represents the number of genes enriched; colour indicates p-value. (**d**) Protein-protein interaction network of Fisetin-associated genes constructed using the STRING database. Nodes represent proteins, and edges represent known or predicted interactions. Central hub genes (including, IL6, SERPINE1, AXL, MET) are densely connected, indicating potential roles in lung cancer signalling

To investigate the molecular mechanisms by which Fisetin exerts anti-cancer effects in lung adenocarcinoma (LUAD), we performed a multi-layered analysis focusing on gene expression, interaction networks, and pathway regulation. Gene expression analysis using GSCALite revealed that several Fisetin-targeted genes exhibited significant subtype-specific differences in LUAD. Among these, IL6, SERPINE1, PDGFA, MMP3, MST1, MET, and ENPP2 showed strong upregulation in LUAD, with adjusted FDR values indicating high statistical significance. Notably, IL6 and SERPINE1 emerged as prominent markers, highlighting their potential role in inflammation-driven oncogenesis within the lung cancer microenvironment (Fig. 13a).

To gain deeper insight into the regulatory roles of individual genes, we examined their pathway-level activities using data from GSCALite. A pathway heatmap analysis showed that genes such as BIRC5, AXL, MMP3, and MET were involved in the activation of EMT and PI3K-AKT signalling pathways, both of which were notably suppressed upon Fisetin exposure. In contrast, pro-apoptotic and tumor-suppressive influences were linked to genes such as MST1 and NOS3, reflecting Fisetin’s dual action in promoting apoptosis while attenuating oncogenic signalling in LUAD (Fig. 13b).

Further, functional enrichment analysis performed through DAVID identified a range of biological processes and signalling pathways significantly associated with the target gene set. These included NF-κB signalling, EGFR and VEGFR pathways, PI3K-Akt, JAK-STAT, apoptosis, extracellular matrix disassembly, and cell migration all of which are integral to lung cancer progression and metastatic potential (Fig. 13c). This enrichment supports the hypothesis that Fisetin’s anticancer activity is mediated through its broad interference with inflammatory and proliferative signalling cascades.

**Fig. 14:**
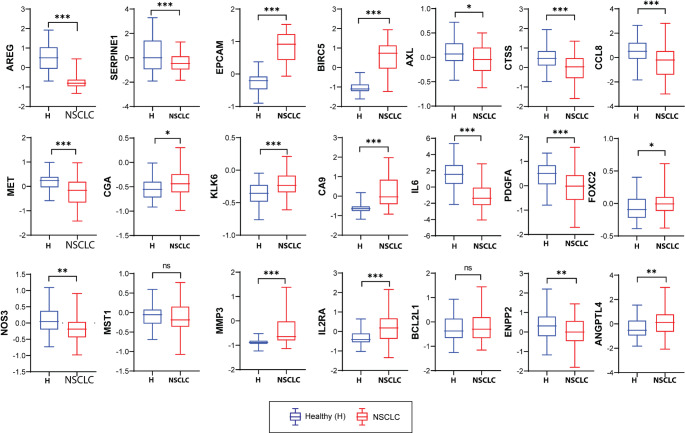
Validation of Fisetin-associated gene expression in NSCLC using GEO dataset GSE19188: Boxplots displaying differential expression of 24 Fisetin-associated genes between healthy lung tissue (H) and non-small cell lung cancer (NSCLC) samples from the GSE19188 dataset. Genes such as SERPINE1, IL6, MMP3, PDGFA, MET, and AXL were significantly overexpressed in NSCLC, supporting their involvement in lung tumorigenesis and potential modulation by Fisetin. Statistical significance: *p* < 0.05(*), *p* < 0.01 (**) ,*p* < 0.001 (***), ns: not significant

To explore the functional interplay among these genes, a protein-protein interaction network was constructed using the STRING database. The resulting network revealed that central nodes such as IL6, SERPINE1, PDGFA, MET, and AXL formed dense interaction clusters with other Fisetin-responsive genes (Fig. 14d). These hubs are well-documented regulators of key oncogenic processes, including angiogenesis, EMT, proliferation, and immune modulation. Their centrality within the network suggests that Fisetin may disrupt tumour-promoting signalling by simultaneously targeting multiple pathways involved in LUAD pathogenesis.

## Validation of Fisetin-Responsive Gene Expression in NSCLC Using GEO Dataset GSE19188

To validate the differential expression of Fisetin-associated genes in lung cancer, we analyzed gene expression profiles from the publicly available microarray dataset GSE19188, which includes lung tumor tissues (NSCLC) and normal lung samples. A comprehensive expression analysis revealed that several key genes previously identified through GSCALite and STRING showed significant upregulation in non-small cell lung cancer (NSCLC) compared to healthy controls. Notably, SERPINE1, AREG, IL6, MMP3, PDGFA, MET, CTSS, CCL8, BIRC5, EPCAM, CA9, AXL, and FOXC2 were significantly overexpressed in NSCLC tissues (*p* < 0.001), reinforcing their relevance in lung tumorigenesis (Fig. 14).

Genes such as NOS3, ENPP2, ANGPTL4, and KLK6 also exhibited moderate yet significant elevation (*p* < 0.01 or *p* < 0.05), whereas MST1 and BCL2L1 showed no statistically significant difference. The consistent overexpression of inflammatory and pro-metastatic genes such as IL6, SERPINE1, and MMP3 across both computational and experimental datasets underscores their potential as key effectors modulated by Fisetin in lung cancer. These findings support the hypothesis that Fisetin’s therapeutic impact on A549 cells may be mediated through suppression of these dysregulated genes in NSCLC.

## Discussion

In the present study, we evaluated the effects of FP, FNE, FME, and a combined FNE/FME treatment on multiple hallmarks of NSCLC, while also examining the regulation of cancer-related proteins and exploring their functional connections through in silico analysis.

The MTT assay determined the minimum concentrations of our treatments to exhibit significant cytotoxicity of A549 cells. In doing so, we confirmed that pure FP did not result in any reduction of cell viability, up to the maximum concentration used, of 20 µg/mL (69.87 µM). Previous studies have noted no impact of pure fisetin on cell viability up to 5.72 ug/mL (20 µM) [47], and so our study confirms that even at higher concentrations, FP does not exhibit cytotoxic effects on A549 cells. Conversely, FNE exhibited cytotoxicity at 10 µg/mL (34.93 µM), supporting the approach of encapsulating poorly soluble bioactive molecules in advance delivery systems to improve their therapeutic efficacy [11]. FME also exhibited higher cytotoxicity than FP, at 10 µg/mL. Furthermore, the FNE/FME mix resulted in a 50% greater effect than either FNE or FME alone, with cytotoxicity at 5 µg/mL (17.46 µM corresponding fisetin concentration). This decrease in cell viability suggests a complementary effect when combining FNE and FME, supporting previous authors’ observations that combining fisetin with other therapeutic agents enhances the therapeutic outcome [47, 57, 58]. However, to identify whether this effect is synergistic or additive would require further investigation with models such as Chou-Talalay or Bliss Independence analysis.

In a previous study assessing the effect of fisetin on colony formation, Adhami et al., focussed on prostate cancer PC3 cells. They reported that fisetin treatment at 20 µM did not significantly inhibit colony formation, which aligns with our current study on A549 cells [59]. However, *Tsai* et al. (2018) found that exposing breast cancer 4T1 and JC cell lines to just 5 µM and 10 µM, resulted in inhibition of colony formation and cell migration [60]. In our present study we confirmed significant inhibition of colony formation of A549 cells was not achieved when treated with FP at 10 µg/mL (35 µM). This suggests that the therapeutic action of fisetin may be dependent on the type of cancer cells and concentration of treatment, warranting further investigation.

The present clonogenic assay produced results consistent with the cytotoxicity obtained with the MTT assay, whereby FP did not significantly inhibit colony formation at the observed concentrations. In contrast, both the FNE and FME significantly reduced colony formation with the FNE resulting in an 87.5% greater effect than the FME. These findings are in agreement with Gao et al. (2025), who reported that colony formation of liver cancer cell lines, HepG2, Huh-7, Hepa1-6 and MHCC97 was inhibited when treated with Fisetin at concentrations of 16µM, 16 µM, 48 µM and 48 µM respectively, then measured 48 h (*p* < 0.001) [61]. Additionally, in our study the FNE/FME Mix had a 100% reduction, implying an augmented effect when combining fisetin with other treatments and is worth further mechanistic investigation.

Cell migration as shown in the Scratch Wound Assay had similar outcomes, whereby the FP did not significantly inhibit cell migration. Unlike the previous assays, FME alone did not yield any significant effect on the wound closure, whereas the FNE resulted in 53% less cell migration compared to control.

The FNE/FME mix provided a further inhibition of cell migration with 73% decrease of wound closure compared to control, and 41% further inhibition than FNE alone. With these results indicating a lack of cytotoxicity displayed by FP, it was removed from the remaining functional assays to focus on FNE, FME and the FNE/FME Mix.

The present study identifies 22 proteins associated with various functions of cancer progression which are impacted by fisetin. The protein array analysis confirms the enhanced therapeutic effect of combining FNE and FME. These proteins have been categorised into functional groups with their relevance in NSCLC tumour development, EMT, the TME, immunomodulation, therapy resistance, and metastasis. Of interest is the regulation of the protein Amphiregulin, a low affinity ligand which promotes sustained EGFR signalling. If unregulated, it will trigger cell proliferation and downstream signalling pathways such as MAPK/ERK and PI3K/AKT [62]. The highest rate of protein inhibition for both FME and the FNE/FME mix was against eNOS, which is associated with inflammation and the formation of cancer-associated fibroblasts (CAFs) within the TME. Furthermore, eNOS and the associated nitric oxide increase the permeability of the tumour-blood barrier, paving the way for tumour cell intravasation and metastasis [63, 64]. Considering that at time of diagnoses, 50% of LC cases have already formed metastases, accounting for 90% of LC mortality, it is imperative to explore the impact of fisetin on these mechanistic pathways. This in conjunction with its role in upregulating inflammatory pathways such as nuclear factor-kB (NF-kB) and cyclooxygenase-2 (COX2), makes eNOS a potential therapeutic target [64].

Previous studies have confirmed the synergistic effect of phytoceuticals and pharmaceutical drugs, enhancing the efficacy of the drug at a lower concentration [45–47, 65]. Separately, it has been established that the plethora of microorganisms which make up the gut microbiome enhance the bioavailability of flavonoids [66]. To our knowledge, ours is the first to investigate fisetin and FME concurrently and, as shown in our results, the combination of FNE and FME results in increased anti-cancer effects compared to the single treatments alone. These data highlight potential targeted treatment [67–71] which is imperative to furthering the development of future impactful therapies [72, 73].

Identifying any augmented effects of various molecular compounds from a single plant extract may be predicted, as we have done, with computational analysis. To improve the connection between our in silico and in vitro findings, it is important to highlight the overlap between the computationally identified fisetin-associated genes and the proteins downregulated in our experimental assays. Our integrative computational analysis elucidates the oncogenic landscape of Fisetin-responsive genes in LUAD, offering mechanistic insights that support the phenotypic effects observed in vitro. Several genes predicted through GSCALite, STRING and DAVID analysis including IL6, SERPINE1, PDGFA, MET, AXL, MMP3 and ENPP2 correspond directly to proteins significantly reduced by FNE, FME or their combination in A549 cells. These molecules play central roles in pathways such as NF-κB signalling, angiogenesis, epithelial mesenchymal transition and extracellular matrix remodelling, all of which were inhibited at the protein level in our array data [21]. This alignment between predicted molecular regulators and experimentally confirmed protein suppression strengthens the biological relevance of our computational analysis and supports the conclusion that fisetin-loaded nanoemulsion and fecal microbiome extract modulate multiple converging oncogenic pathways.

Our in silico analysis also reinforces the mechanistic patterns observed in the protein array. The enriched pathways identified including NF-κB, PI3K–Akt, JAK–STAT, VEGF, EGFR and EMT-associated signalling directly overlap with the protein-level changes induced by FNE, FME and their combination. Several of the downregulated proteins, such as IL6, AXL, MET, MMP3 and PDGFA, are key components of these pathways. This convergence between pathway enrichment and experimental protein suppression strengthens the mechanistic interpretation of our findings and supports the conclusion that fisetin-loaded nanoemulsion and fecal microbiome extract act on multiple interconnected oncogenic.

Building on these insights, we next examined whether these computationally identified genes showed similar patterns of dysregulation in patient-derived samples. To validate these findings, we analysed gene expression profiles from the GEO dataset GSE19188, comparing NSCLC tissues with normal lung samples. This revealed a consistent overexpression of several fisetin-associated genes, including IL6, SERPINE1, AXL, MET, MMP3 and PDGFA, corroborating both computational predictions and the protein-level reductions observed in vitro. The convergence of these datasets underscores the clinical relevance of these targets in LUAD and suggests that fisetin may attenuate tumour progression by downregulating pro-metastatic and pro-inflammatory mediators. Taken together, this validation layer supports the hypothesis that fisetin exerts its anti-cancer effects through multi-targeted inhibition of LUAD-associated signalling networks and highlights key genes that warrant further functional investigation as potential therapeutic targets.

Despite the promising results of the present study, the in vitro experiments were using a two-dimensional cell culture model and therefore further investigations can be performed using a three-dimensional, multicellular tumour *milieu* employing a tumour organoid model and in vivo studies. Additionally, while various microorganism strains have been identified in the current FME, the microbiome is a highly complex ecosystem with in excess of 2000 species, yet current sequencing methods are limited to identifying several hundred at a time [74]. Additionally, our current data does not allow for differentiation of the actions of the organisms themselves, or the metabolites they produce such as butyric acid, acetic acid and propionic acid [43, 75], and this could be the focus of future research.

## Conclusions and Future Directions

The findings of this study demonstrate a promising therapeutic avenue involving the co-administration of FNE and FME in attenuating multiple hallmarks of NSCLC. Functional in vitro assays confirmed that this combination exerts enhanced inhibitory effects on cell viability, colony formation, and migration, compared to fisetin powder or either agent alone. These anti-cancer effects were further supported by human oncology protein array data, which revealed significant downregulation of multiple proteins implicated in oncogenesis, epithelial-mesenchymal transition (EMT), angiogenesis, immunomodulation, therapeutic resistance and metastasis. Although beyond the scope of the present study, future investigations that validate the downregulated oncogenic markers using RT-qPCR would represent valuable follow-up approaches.

Ongoing work within this project will further elucidate the molecular mechanisms underlying the enhanced anti-cancer effects observed with the FNE/FME combination. Furthermore, identifying whether this effect is synergistic or additive utilising Chou-Talalay or Bliss Independent modelling is warranted [76, 77]. Previous studies have established a robust precedent, demonstrating that co-administration of fisetin with other phytoceuticals can produce measurable synergistic effects. Co-administration of fisetin with quercetin and separately, fisetin with naringenin, in breast cancer cell lines (MCF-7, MDA-MB-231, T47D, 4T1) produced combination index (CI) values below 1, confirming strong synergism in reducing cell proliferation, migration, and colony formation [78, 79] This approach is strengthened by the growing recognition that fisetin synergizes with pharmacological agents such as paclitaxel in A549 lung carcinoma cells (CI ≈ 0.15 at 10 µM Fisetin + 0.1 µM Paclitaxel), underscoring its potential to amplify therapeutic efficacy through combination strategies [47]. These findings collectively support that flavonoid–flavonoid and flavonoid-pharmaceutical combinations can potentiate anticancer outcomes through complementary molecular mechanisms.

Building upon this foundation, the present investigation exploring positive interactions between FNE and FME aims to extend this paradigm, specifically by coupling a bioactive polyphenol with a microbiome-derived component to assess dual modulation of oncogenic pathways. Collectively, these published synergistic models provide a strong scientific rationale for examining the combined activity of FNE and FME, hypothesizing that similar augmentation may emerge from the interplay between plant-based therapeutics and microbiome metabolites.

This multi-targeted strategy, rooted in nanomedicine and microbiome modulation, represents an emerging frontier in NSCLC therapeutics. Future investigations should prioritize in vivo validation and metabolomic profiling of FME. In particular, in vivo studies and three-dimensional tumour models are necessary to validate the current findings, identify microbiome mediated mechanisms and to act as a benchmark for future clinical research and product development. Additionally, given the role of the gut microbiome in modulating drug bioavailability, further characterization of the active metabolites within the FME could provide insights into potential synergies with other anti-cancer agents. Investigations into combination strategies involving phytoceutical nanoemulsions, microbiome extracts, and chemotherapy drugs may also yield promising avenues for overcoming treatment resistance. Ultimately, these findings pave the way for more targeted and effective therapeutic strategies, bridging the gap between natural compounds, nanomedicine, and microbiome research in the fight against non-small cell lung cancer, providing a new direction to lung cancer clinics.

## Data Availability

Data is available from the Corresponding Author upon reasonable request.
